# The admixture of *Quercus* sp. in *Pinus sylvestris* stands influences wood anatomical trait responses to climatic variability and drought events

**DOI:** 10.3389/fpls.2023.1213814

**Published:** 2023-11-16

**Authors:** Giulia Silvia Giberti, Georg von Arx, Alessio Giovannelli, Ben du Toit, Lucrezia Unterholzner, Kamil Bielak, Marco Carrer, Enno Uhl, Felipe Bravo, Giustino Tonon, Camilla Wellstein

**Affiliations:** ^1^ Faculty of Agricultural, Environmental and Food Sciences, Free University of Bolzano - Bozen, Bolzano, Italy; ^2^ Swiss Federal Institute for Forest, Snow and Landscape Research WSL, Birmensdorf, Switzerland; ^3^ Oeschger Centre for Climate Change Research, University of Bern, Bern, Switzerland; ^4^ Istituto di Ricerca sugli Ecosistemi Terrestri (IRET), Consiglio Nazionale Ricerche, Sesto Fiorentino, Italy; ^5^ National Biodiversity Future Center (NBFC), Palermo, Italy; ^6^ Department of Forest and Wood Science, Faculty of AgriSciences, Stellenbosch University, Stellenbosch, South Africa; ^7^ Department of Land Environment Agriculture and Forestry, University of Padova, Legnaro, Italy; ^8^ Chair of Forest Growth and Woody Biomass Production, Technische Universität Dresden, Tharandt, Germany; ^9^ Department of Silviculture, Institute of Forest Sciences, Warsaw University of Life Sciences-SGGW, Warsaw, Poland; ^10^ School of Life Sciences, Chair for Forest Growth and Yield Science, Technical University of Munich (TUM), Freising, Germany; ^11^ Bavarian State Institute of Forestry (LWF), Freising, Germany; ^12^ Instituto Universitario de Investigación en Gestión Forestal Sostenible (iuFOR). Escuela Técnica Superior de Ingenierías Agrarias de Palencia, Universidad de Valladolid, Palencia, Spain

**Keywords:** climate change, drought, inter-specific facilitation, mixed forest, *Pinus sylvestris*, quantitative wood anatomy

## Abstract

**Introduction:**

Forests are threatened by increasingly severe and more frequent drought events worldwide. Mono-specific forests, developed as a consequence of widespread management practices established early last century, seem particularly susceptible to global warming and drought compared with mixed-species forests. Although, in several contexts, mixed-species forests display higher species diversity, higher productivity, and higher resilience, previous studies highlighted contrasting findings, with not only many positive but also neutral or negative effects on tree performance that could be related to tree species diversity. Processes underlying this relationship need to be investigated. Wood anatomical traits are informative proxies of tree functioning, and they can potentially provide novel long-term insights in this regard. However, wood anatomical traits are critically understudied in such a context. Here, we assess the role of tree admixture on *Pinus sylvestris* L. xylem traits such as mean hydraulic diameter, cell wall thickness, and anatomical wood density, and we test the variability of these traits in response to climatic parameters such as temperature, precipitation, and drought event frequency and intensity.

**Methods:**

Three monocultural plots of *P. sylvestris* and three mixed-stand plots of *P. sylvestris* and *Quercus* sp. were identified in Poland and Spain, representing Continental and Mediterranean climate types, respectively. In each plot, we analyzed xylem traits from three *P. sylvestris* trees, for a total of nine trees in monocultures and nine in mixed stands per study location.

**Results:**

The results highlighted that anatomical wood density was one of the most sensitive traits to detect tree responses to climatic conditions and drought under different climate and forest types. Inter-specific facilitation mechanisms were detected in the admixture between *P. sylvestris* and *Quercus* sp., especially during the early growing season and during stressful events such as spring droughts, although they had negligible effects in the late growing season.

**Discussion:**

Our findings suggest that the admixture between *P. sylvestris* and *Quercus* sp. increases the resilience of *P. sylvestris* to extreme droughts. In a global warming scenario, this admixture could represent a useful adaptive management option.

## 1 Introduction

Forest vulnerability and dieback induced by global warming and drought events are increasing worldwide (Allen et al., 2010; Allen et al., 2015). Drought severity and frequency are expected to increase in the next decades (Dai et al., 2018), and Central and Southern Europe are predicted to be strongly affected (Giorgi, 2006; Somorowska, 2022).

An increasing number of studies have consistently documented higher productivity (Del Río and Sterba, 2009; Bielak et al., 2014) and greater resilience to global climate changes in mixed forests versus monocultures (e.g., Pretzsch et al., 2013b; Pardos et al., 2021). The functional benefits derived from tree species diversity rely on the niche complementarity hypothesis, which states that diversity promotes inter-specific facilitation by enhancing access to resources via niche differentiation (Tilman, 1999; Zhang et al., 2012; Pretzsch et al., 2013b). It could be expected that individuals belonging to different species and having dissimilar resource needs show lower competition in comparison with the intra-specific competition among individuals within monocultures. In addition, in mixed forests, niche complementarity could improve growth and increase overall ecosystem resilience. It is known that benefits of increased biodiversity on tree performance vary in space, time, and with different species (Forrester, 2014). Tree species-specific vulnerabilities against various kinds of stressors (i.e., pests, windstorms and drought) are less severe in mixed-forest ecosystems, making them more resilient to perturbations. Studies on species interactions in mixed forests showed contrasting results in terms of growth/productivity and resilience to drought events. As an example, in the works of Bielak et al. (2014) and Pretzsch et al. (2015), the admixture of species increased tree productivity compared with pure stands. In the work of Zeller et al. (2017), the increase in productivity occurred only for one species (*P. sylvestris*), whereas the opposite was shown for the other (*Fagus sylvatica*). Non-significant differences in productivity between mixed and pure forests were reported by Giberti et al. (2022). Pretzsch et al. (2013b) showed that, in mixed- forest tree, resilience to drought is affected by species-specific interactions and that different reactions to drought can be observed depending on the species considered. Similar results were presented by Bello et al. (2019) and Thurm et al. (2016), who found beneficial effects of tree admixture during drought events for *Q. petraea* and *Pseudotsuga menziesii*, but not for *P. sylvestris* and *F. sylvatica*. Instead, Merlin et al. (2015) did not find significant effects of tree admixture on tree resistance and resilience to drought when considering mixed *P. sylvestris* –*Q. petraea* stands. According to Callaway and Walker (1958), benefits of increased biodiversity could be more apparent under harsher conditions such as under resource limitation. This hypothesis was supported by Paquette and Messier (2011), who showed the beneficial effects of complementarity to be more important in stressful environments (boreal forest), rather than in more stable environments (temperate forest), and by Pretzsch et al. (2013b), who showed that facilitation effects related to mixed-forest composition emerged during episodic stressful events such as drought. These documented experiences demonstrate that tree performance in mixed forests can vary greatly depending on specific conditions and further studies are required to assess the influence of different species under variable soil and climate conditions.

Common parameters adopted to understand whether mixed forests can be more productive and/or resilient than pure forests include direct measures of tree growth, i.e., ring width, basal area increment, and derived measures such as volume increment per hectare (Del Río and Sterba, 2009; Michelot et al., 2012; Pretzsch et al., 2013a; Pretzsch et al., 2013b; Merlin et al., 2015; Steckel et al., 2019). These proxies are only related to tree growth and do not allow inferences on possible trade-offs occurring among functional tree ring properties. The assessment of xylem traits can provide a higher-resolution insight into the processes that relate to tree resilience. Physiological parameters such as isotopic signature, predawn leaf water potential, and wood density were scarcely explored in the context of investigating tree resilience under climatic alterations in mixed forests (Toïgo et al., 2015; Zalloni et al., 2018; Bello et al., 2019; Giberti et al., 2022). The influence of tree admixture on xylem traits has not been explored yet, although xylem trait variability provides information about physiological processes during acclimation, in response to changing abiotic and biotic conditions (Fonti et al., 2010; Cuny et al., 2014; Pellizzari et al., 2016; Pacheco et al., 2018a; Castagneri et al., 2020; Guérin et al., 2020).

*Pinus sylvestris* L. (Scots pine) is a pioneer, light-demanding evergreen species that is of great ecological and economic importance. It is one of the most widely distributed Eurasian pine species, occurring under a variety of different environmental conditions, showing high genetic variability, high ecological plasticity (Cheddadi et al., 2006), and local acclimation to climate change (Martínez-Vilalta et al., 2009; Martín et al., 2010; Matisons et al., 2019). In recent times, mortality and decline of *P. sylvestris* induced by drought were reported across its southern distribution (Martínez-Vilalta and Piñol, 2002; Eilmann et al., 2009; Allen et al., 2010; Hereş et al., 2014; Pellizzari et al., 2016; Petrucco et al., 2017). On the basis of current climate projections, a further significant decline in Central Europe is being forecasted (Buras and Menzel, 2019). The admixture between *P. sylvestris* and *Quercus* sp. has been investigated focusing on complementarity in water uptake (Bello et al., 2019), in growth and yield (Del Río and Sterba, 2009; Merlin et al., 2015; Pretzsch et al., 2020), and in wood density (Toïgo et al., 2015; Giberti et al., 2022). Inter-specific facilitation mechanisms occur due to the complementary realized by differences in leaf phenology, wood anatomy (Michelot et al., 2012), and root stratification and morphology (Merlin et al., 2015; Bello et al., 2019), with *P. sylvestris* having a shallow root system (0 cm to 40 cm) and a strong first taproot (Merlin et al., 2015) and *Quercus* sp. having a generally deeper root system. The two species show different seasonal growth patterns and different strategies for coping with drought (Eilmann et al., 2009; Merlin et al., 2015; Bello et al., 2019). These characteristics could provide an overall reduction in competition during drought events. Because the inter-specific interactions can change depending on the growing season, a careful assessment of the extent and the timing of drought is fundamental to forecasting the response of mixed forests to extreme droughts that are likely to occur more frequently in the next decades (Merlin et al., 2015).

In this study, we analyze the effect of tree admixture on *P. sylvestris* xylem traits under two different climatic conditions: 1) Continental (Poland), which has optimum climatic conditions for *P. sylvestris* and lies in the core of the native range of this species (Caudullo et al., 2017); and 2) Mediterranean (Spain), at the southern edge of *P. sylvestris* distribution range (Caudullo et al., 2017). At both sites, we identified pure *P. sylvestris* forests of local provenance as well as mixed forests of *P. sylvestris* and *Quercus petraea* L. (Poland) and *Quercus pyrenaica* WILLD (Spain). *Quercus petraea* is partially shade- tolerant, well adapted to occasional droughts. *Quercus pyrenaica* is drought-tolerant; under transitional climatic zones, between sub-humid temperate and semi-arid Mediterranean, it is adapted to shade (Caudullo et al., 2017).

The following xylem traits were selected for this study as they are likely candidates to elucidate physico-mechanical xylem response mechanisms to climatic factors such as drought events:

i) Mean hydraulic diameter (DH), for the assessment of tree hydraulics, because according to Hagen-Poiseuille law, the conductivity of the hydraulic conduit is directly proportional to its radius raised to the fourth power (Zimmermann, 2013). The wider the conduits, the more efficient the hydraulic system, but the more prone it putatively becomes to cavitation, which could eventually lead to drought-induced dieback (Eilmann et al., 2009; Desoto et al., 2011; Bryukhanova and Fonti, 2013).
ii) Cell wall thickness (CWT; averaged over all walls) related to mechanical strength preventing cell collapse (Hacke et al., 2001) followed by cavitation and to the amount of carbon allocated to the xylem (Cuny et al., 2015).
iii) Anatomical wood density {AD; i.e., cell wall area/[cell wall area + lumen area (LA)]} as proxy for absolute wood density (Björklund et al., 2017; Björklund et al., 2021).

Trees in mixed forests could have greater access to environmental resources, which can buffer and help trees to endure stressful events such as drought (Magruder et al., 2013; Pretzsch et al., 2013b; Thurm et al., 2016). Hence, we expect *P. sylvestris* in mixed forests to show reduced climate sensitivity (enhanced climatic resistance/resilience), thereby presenting a wood architecture with no or only minor xylem trait adjustments induced by drought stress. On the contrary, *P. sylvestris* growing in pure stands could show higher climate sensitivity, thus presenting specific xylem trait adjustments induced by drought stress. Trees growing in semi-arid conditions are characterized by an inconstant growth rate due to regularly occurring drought stress (Biondi and Qeadan, 2008). Hence, we also expect to find generally higher xylem trait variability in *P. sylvestris* trees in the Spanish site compared with that in the Polish site. In addition, trees under drought stress tend to show high xylem trait variability (Levanič et al., 2011; Hereş et al., 2014; Guérin et al., 2020; Buras et al., 2023), and we expect that pines growing in pure stands could present this feature more strongly.

More specifically we hypothesize the following.

1. At both sites, xylem traits of *P. sylvestris* in pure forests will show higher sensitivity to climate (i.e., temperature, precipitation, and aridity index) and also higher variability compared with *P. sylvestris* growing in mixed forests. As response to drought, pure *P. sylvestris* xylem morphology will show reduced ring width, reduced DH, and increased CWT and AD. In *P. sylvestris* trees growing in mixed stands, these traits will present reduced or none xylem trait adjustments to drought events.
2. Under Mediterranean climate, summer drought occurs naturally, and we expect *P. sylvestris* to show higher xylem traits variability compared with *P. sylvestris* grown under continental climate.

## 2 Materials and methods

### 2.1 Study areas, forest, and species characteristics

**Figure 1:**
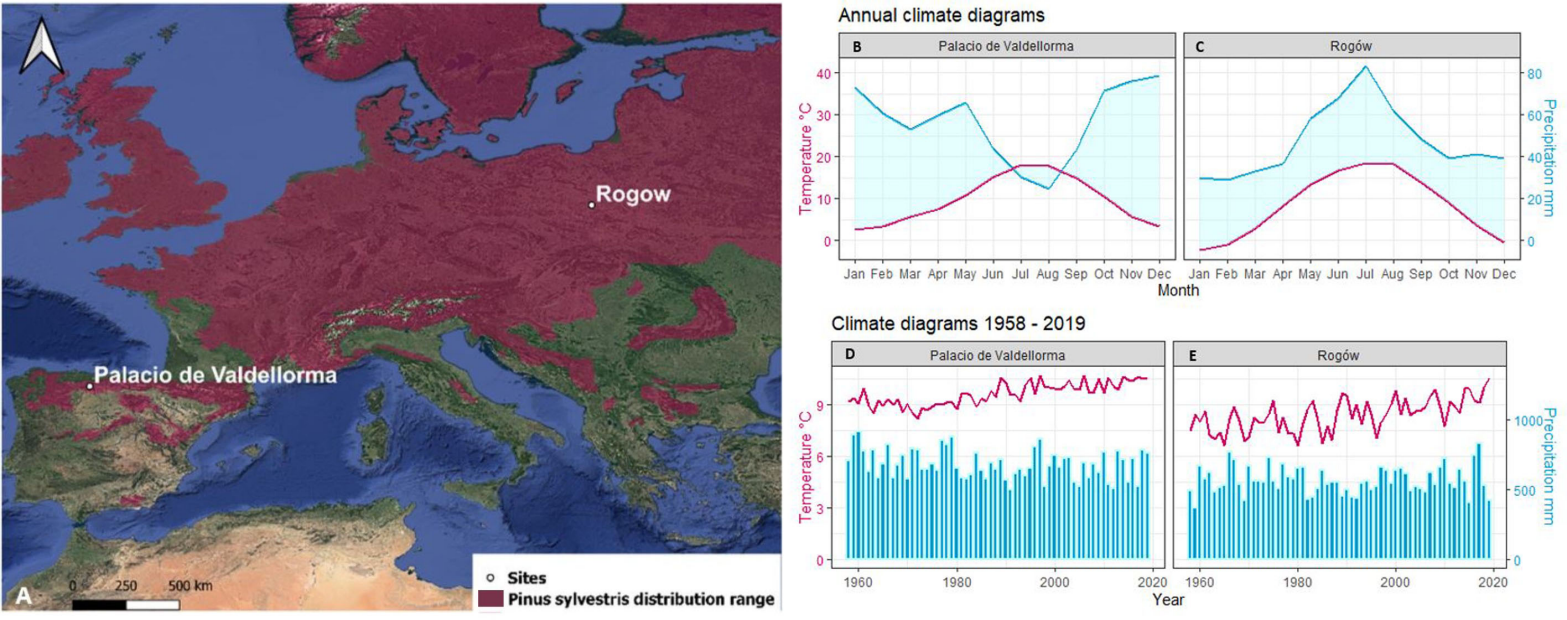
**(A)** Site location: Rogòw, 51°48′42.30″N 19°49′55.55″E, 200 m a.s.l., Palacio de Valdellorma 42°48′54.50″ N, 5° 6′45.64″ W, 916 m a.s.l. on distribution range of *Pinus sylvestris* (Caudullo et al., 2017). Relative annual climatogram: **(B)** Palacio de Valdellorma and **(C)** Rogów; climatogram for the time span 1958–2019: **(D)** Palacio de Valdellorma and **(E)** Rogów. The climatic records were obtained from the local weather station for Rogów and from the Terraclimate dataset for Palacio de Valdellorma.

The first study site was in Poland, at the Rogów Forest Experimental Station (51°48′42.30 ″ N, 19°49′55.55 ″ E, 200 meter above the sea level (m a.s.l.)), situated 100 km southwest of Warsaw, managed by the Warsaw University of Life Sciences-SGGW (**Figure 1**). Soils are silty and sandy loams developed over sandstone bedrock and are classified as Luvisols (Anjos et al., 2014). Following Köppen’s classification, the climate is Dfb, i.e., “warm-summer humid continental” (Beck et al., 2018). The site includes a pure *P. sylvestris* forest and a mixed one with *P. sylvestris* and *Q. petraea.* Pine trees were sowed in 1936 and in 1944 after clearcutting an oak stand, whereas oak trees regenerated naturally. The site did not undergo significant disturbance by wind storms or pests. The second site was in Spain, at Palacio de Valdellorma (42°48′54.50″ N, 5° 6′45.64″ W, 916 m a.s.l.), in León province, in an experimental mixed forest managed by the Sustainable Forest Management Research Institute, Universidad de Valladolid (**Figure 1**). Soils are Humic loamy Cambisols, with an impermeable layer below 150 cm (Zádorová et al., 2021). Following Köppen’s classification, the climate is Csb, i.e., “warm-summer Mediterranean” (Beck et al., 2018). The site hosted a pure *P. sylvestris* and a mixed *P. sylvestris* and *Q. pyrenaica* forest. Pine trees were sown in 1971–1972 following a partial clear-cut of pre-existing oak trees.

#### 2.1.1 Pinus sylvestris

The Central European *P. sylvestris* is a fast growing high-performance species reaching considerable height at maturity (Wachowiak et al., 2018). Being evergreen, able to photosynthesize during warm winters (Michelot et al., 2012), it benefited from recent-winter and late winter temperature increases. Indeed, late- winter and early- spring temperatures are considered among the main drivers supporting *P. sylvestris* development across Central Europe (Koprowski, 2012; Misi and Náfrádi, 2016; Misi et al., 2019; Giberti et al., 2022), where it showed vulnerability to water deficit during the early growing season (Michelot et al., 2012). At this latitude, budburst usually occurs by mid-May (Perot et al., 2021). At lower latitudes, *P. sylvestris* tend to slow growth, to reduce height, and to establish earlier budset and budburst compared with central European populations (Wachowiak et al., 2018). Under Mediterranean climate, an extended growing season was reported, with xylem cell production from the mid of April until late October (Camarero et al., 2011). Under these climatic conditions, spring rains always positively influenced pine growth, whereas drought reduced it (Bogino et al., 2009; Camarero et al., 2011).

#### 2.1.2 *Quercus petraea* and *Quercus pyrenaica*

Being ring-porous, the growth of these oak species is not regular during the growing season: in springtime, they grow fast and form wide earlywood (EW) cells (González-González et al., 2013) but tend to be highly sensitive to spring drought (Merlin et al., 2015). *Quercus petraea* is a mesophilic oak, well adapted to drought events (Merlin et al., 2015). In Central Europe, it shows xylem cell production before budburst (Michelot et al., 2012), which usually occurs around early to mid-April (Perot et al., 2021; Gafenco et al., 2022), whereas complete leaf unfolding occurs in late April (Gafenco et al., 2022). *Quercus pyrenaica* is more drought-tolerant than the former species. At similar latitude and conditions to the Spanish site, cell production in *Q. pyrenaica* starts in mid-March, prior to budburst, which occurs a few weeks later (Pérez-de-Lis et al., 2016), and leaf expansion is generally completed in May but can eventually last until late June. The last EW cell forms by the end of June, and latewood (LW) production stops in mid-September, even if cell lignification continued for one more month (González-González et al., 2013). Drought- tolerant oaks (anisohydric) tend to show a contrasting water-use strategy in comparison with drought- avoiding (isohydric) pine; this different intra-annual drought sensitivity may produce contrasting drought response.

### 2.2 Climatic data

Polish climatic data were obtained from two weather stations located in the proximity: Łódź, at 38 km west, and Skierniewice, at 20 km northeast of the study site. The data were assembled by applying an inversely weighted mean by distance to monthly data derived from the two weather stations covering the period 1952–2019. No weather stations were in close proximity to the Spanish site; monthly climatic data were extracted from the online dataset Terraclimate (Abatzoglou et al., 2018), a 4- km-gridded, high-resolution dataset designed to calculate water balance, available for the time span 1958 –2019. For consistency, the correlation between Polish weather station climatic records and the ones of the corresponding 4-km grid cell (centered in 51.8125 N, 19.8542 E) available for the Polish site in Terraclimate were assessed by monthly correlation analysis for the period 1958– 2019. The majority of the climatic variables showed high correlations (R > 0.90).

Rogów is characterized by a mean annual temperature of 8.3°C and an annual precipitation of 565 mm. The study site climatogram covering the time span 1958 –2019 (**Figure 1**) showed a consistent linear increase in mean annual temperature over time (*p*< 0.001), observed especially during spring and summer months (Chojnacka-Ożga and Ożga, 2018). Precipitation did not show clear trends over time, but it tended to increase during spring. The climate in Palacio de Valdellorma is characterized by a mean annual temperature of 9.2°C and annual precipitation of 679 mm. For the time span 1958 –2019, mean annual temperature showed a linear increase over time (*p<* 0.001), and annual precipitation a linear decrease (*p<* 0.03).

**Figure 2:**
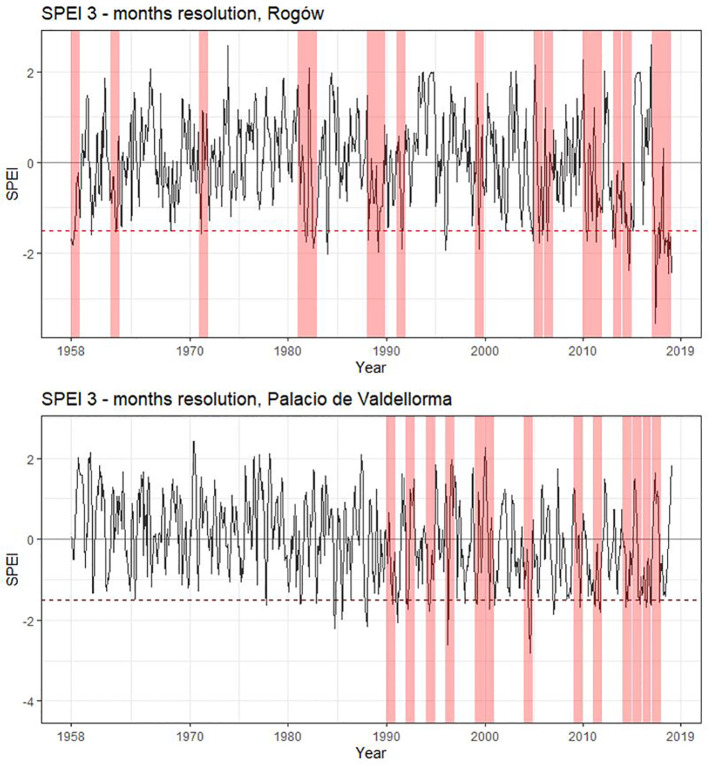
Standardized Precipitation Evapotranspiration Index for the two study sites. SPEI calculated with a 3- month window starting from March to October. Drought years (SPEI< −1.5) are highlighted in pink. Rogów: 1959, 1963, 1972, 1982, 1983, 1989, 1990, 1992, 2000, 2006, 2007, 2011, 2012, 2014, 2015, and 2018. Palacio de Valdellorma: 1991, 1993, 1995, 1997, 2000, 2001, 2005, 2010, 2012, 2015, 2016, 2017, and 2018.

The following climatic variables were considered: monthly precipitation and temperature and the Standardized Precipitation Evapotranspiration Index (SPEI), a multi-scalar index for estimating drought magnitude with respect to normal conditions. SPEI was calculated with R package “SPEI” (Beguería et al., 2017), using a 3- and a 6-month window because they show the highest correlations based on an initial screening. Years characterized by severe drought were identified using SPEI at 3-month resolution<− 1.5 (Potop et al., 2014, **Figure 2**).

### 2.3 Sampling design and lab work

At both sites, we selected six pre-existing plots (three in pure forest and three in mixed forest) close to each other. The plots were flat, homogeneous in terms of altitude, exposition, climate, and soil characteristics. From every plot, we selected six dominant and healthy *P. sylvestris* trees, i.e., the tallest trees with large diameter at breast height, thus having comparable social class and excluding other sources of variation except for climate and tree competition given by tree density and different tree species. Overall, we cored 36 trees per site in pure and the same amount in mixed stands. In mixed forest, we selected trees surrounded only by *Q. petraea* and *Q. pyrenaica*, in Poland and Spain, respectively. The characteristics of the plots are summarized in **Table 1**. The Stand Density Index (calculated with Reineke formula, Reineke, 1933), was employed to determine stand density. This index showed a similar competition level in both forest types in the Polish site (741 in pure forest and 742 in mixed forest) but a different competition level for the two forest types in the Spanish site (935 in pure forest and 1,413 in mixed forest). We collected one core of 1 cm diameter at 1 m height from the northern side of every study tree using an increment borer.

**Table 1:** Tree admixture and plot characteristics for both sites, Rogów and Palacio de Valdellorma.

Site	Rogów						Palacio de Valdellorma					
Forest types	Mixed			Pure			Mixed			Pure		
Plot ID	MPA	MPB	MPC	PPD	PPE	PPF	MPA	MPB	MPC	PPD	PPE	PPF
Plot size, ha	0.2	0.2	0.18	0.12	0.12	0.09	0.2	0.2	0.2	0.04	0.04	0.04
No. of all trees/oaks ha^−1^	610/345	655/365	505/115	708	458	444	5,580/4,245	4,635/3,045	4,370/2,960	2,200	2,350	2,025
BA of all trees/oaks, m^2^ ha^−1^	36/23	42/23	41/10	43	41	38	31/13	32/10	33/13	67	69	57
Stand Density Index	696	790	737	825	721	681	935	932	938	1460	1510	1269
DBH (cm), *Pinus sylvestris*	31.4 ± 4.28	29.6 ± 6.58	31.6 ± 5.81	30.6 ± 5.56	33.8 ± 5.44	34.3 ± 6.15	11.9 ± 4.5	12.5 ± 3.9	12.5 ± 4.7	19.4 ± 5.4	17.7 ± 4.3	18.2 ± 4.3
H (m), *Pinus sylvestris*	24.5 ± 1.69	25.6 ± 1.65	27.4 ± 2.52	26.1 ± 1.38	30.3 ± 1.71	27.9 ± 2.08	13.2 ± 1.0	10.5 ± 1.0	14.1 ± 0.74	12.5 ± 1.0	11.3 ± 1.5	11.1 ± 1.6
DBH (cm), *Quercus* sp.*	28.1 ± 7.66	27.8 ± 5.64	31.1 ± 8.32	NA	NA	NA	5.6 ± 2.9	6.00 ± 2.7	6.27 ± 3.8	NA	NA	NA
H (m), *Quercus* sp.*	24.4 ± 1.22	25.1 ± 1.64	24.3 ± 3.05	NA	NA	NA	NA	NA	NA	NA	NA	NA

*Quercus petraea for Polish site and *Quercus pyrenaica* in the case of the Spanish site.

MP, mixed plot; PP, pure plot. The third letter (A, B, C, D, and F) identifies every plot individually. BA, basal area increment; DBH, diameter at breast height (cm); H, height of the trees (m). DBH and H values are presented with mean ± standard deviation. NA, not available.

The cores were analyzed at the Technical University of Munich Chair for Forest Growth and Yield Science, with LIGNOSTATION™, a high- frequency densitometry assessment instrument, which allows for non-destructive and quick measure of ring width and ring density (Schinker et al., 2003; Zeller et al., 2017). All ring series were cross-dated with TSAP-Win software (Rinntech®, 2010).

**Figure 3:**
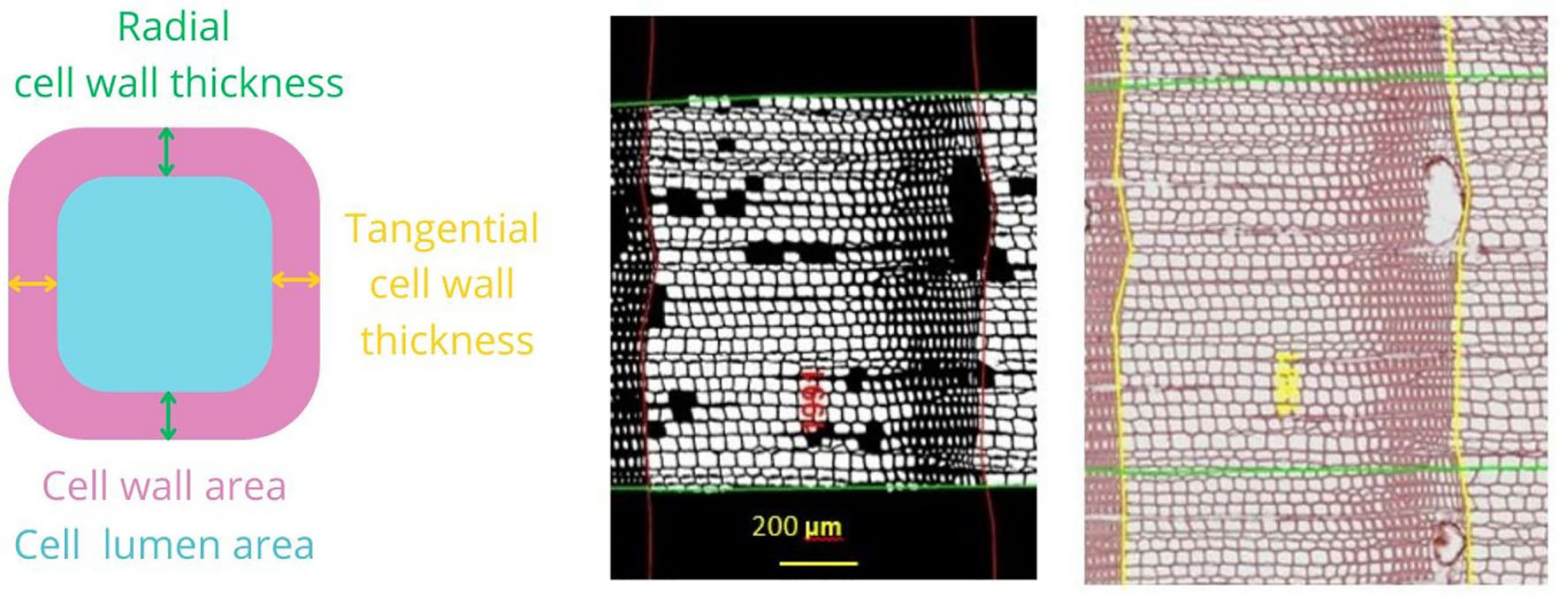
Illustration of anatomical measurements. At the right an image of a stained section where ring borders are drawn (yellow) and calendar years are assigned using ROXAS. The green area represents the area of interest where wood anatomical traits were analyzed. At the middle, there is a black-and-white image where only the cells used in the analysis are highlighted. Scale bar, 200 μm. At the left, there is a schematic representation of the wood anatomical parameters selected for the analysis. Mean hydraulic diameter (DH) = sum (cell hydraulic diameter ^5^)/sum (cell hydraulic diameter ^4^); cell wall thickness (CWT) = (radial cell wall thickness + tangential cell wall thickness)/2; anatomical wood density (AD) = cell wall area/(cell wall area + lumen area).

We selected a subsample of three trees per plot, whose cores showed no signs of damage, for a total of 18 cores per site (nine in pure and nine in mixed forest). The characteristics of the selected trees and the corresponding cores are summarized in **Table 2**. The cores were divided into 4 cm-long pieces soaked in hot water to soften the wood and to remove resin before producing 15 μm -thick transverse sections with a rotary microtome (HistoCore MULTICUT, Leica Biosystems, Nussloch, Germany). The sections were stained with a solution of safranine and astrablue (1% and 0.5% in distilled water, respectively), rinsed with distilled water and then with increasing ethanol concentrations: 50% –96%, and mounted permanently on glass microscope slides with Eukitt (BiOptica, Milan, Italy) following von Arx et al. (2016). High-quality images were obtained with a slide scanner at 100× magnification and 1.99 pixels μ^–1^ resolution (D-sight 2.0 System, Menarini Diagnostics, Florence, Italy). The images were processed with ROXAS v. 3.0.454, a software that allows for quantitative investigation of wood anatomical traits (von Arx and Carrer, 2014; Prendin et al., 2017, **Figure 3**). In total, 3.1 million tracheids (2.0 × 10^6^ for Poland, 1.1 × 10^6^ for Spain) were measured. To avoid redundancy and to select more informative xylem traits, cross-correlation analysis was performed on the following traits: LA, DH, average CWT and AD. Given the high correlation between LA and DH, radial cell wall thickness, tangential cell wall thickness, and the different cell wall measurements (R^2^ > 0.97), we retained only DH, CWT, and AD. Wood anatomical trait chronology was visually compared with the chronology previously obtained with LIGNOSTATION™ for a careful assessment of the calendar years.

**Table 2:** Characteristics of the trees and the cores selected for wood anatomical analyses summarized per forest type at the two sites, in Rogów and Palacio de Valdellorma.

Site	Rogów		Palacio de Valdellorma	
Forest types	Mixed	Pure	Mixed	Pure
Time Span	1948– 2018	1952– 2018	1989– 2019	1979– 2019
DBH (cm)	37.18 ± 6.53	41.41 ± 3.22	22 ± 2.46	23.66 ± 2.18
H (m)	27.17 ± 2.17	29.48 ± 0.989	12.56 ± 1.37	12.94 ± 1.33
Avg series length (years)	55.5	52.2	36.17	23.88
Mean raw ring width (mm)	2.04 ± 1	2.30 ± 1.07	2.57 ± 1.06	2.57 ± 1.36

DBH, diameter at breast height (cm); H, height of the trees (m). DBH and H values are presented with mean ± standard deviation. The number of trees per each forest type is 9.

### 2.4 Ring width and wood anatomical trait analyses

The analyses were performed with R v. 4.0.2 (R: The R Project for Statistical Computing, 2020) using the packages dplyr (Wickham et al., 2023) and dplR (Bunn et al., 2023). Each ring was divided into 100 equal-width tangential sectors, based on the relative position of each cell in the ring (Pacheco et al., 2018b). The intra-annual profile of the wood anatomical traits was carefully inspected; sometimes, EW cells were present in LW and vice versa, due to some bad quality images. On the basis of conduit size, we identified and removed from the dataset the outliers related to those cells. Further outliers were identified in EW and LW sectors, respectively, when the data resulted greater than the Upper Quartile (75th percentile of data distribution) multiplied by 1.5 or lower than the Lower Quartile (25th percentile of data distribution) multiplied by 1.5.

#### 2.4.1 EW-LW anatomical chronologies and relationship with climate

**Figure 4:**
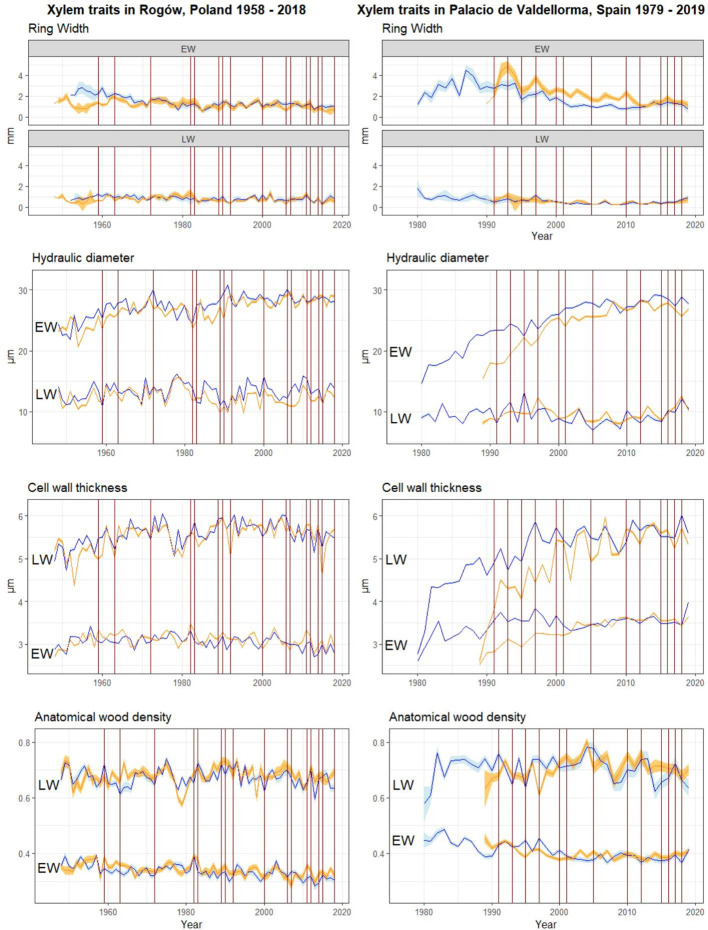
Raw chronologies of EW and LW traits. Orange lines, *P. sylvestris* in mixed forest; blue lines, *P. sylvestris* in pure forest. From top to bottom: EW and LW width, mean hydraulic diameter, cell wall thickness, and anatomical wood density. The vertical red lines indicate the years when drought occurred (cf. **Figure 2**). On the left panel, *P. sylvestris* in Rogów, Poland. On the right panel, *P. sylvestris* in Palacio de Valdellorma, Spain.

To explore the relationship between wood anatomical traits and climate, each sector was assigned to EW (mainly related to water transport functions) or LW (mainly related to mechanical support functions) based on Mork’s Index (Denne, 1989). Median EW and LW values were calculated for each individual tree. The median was preferred to the mean to reduce the impact of skewed parameter distributions. The raw chronologies are presented in **Figure 4**. The age-related trend that characterizes the EW and LW anatomical series was removed using a detrending procedure. This technique accounts also for the axial widening of wood anatomical traits, thus allowing for a comparison between traits collected at different distances from the apex (Petit et al., 2022), as in our case (**Table 2**). Each series was fitted with a cubic smoothing spline with 50% frequency –response cutoff of 30 years. Detrended series were calculated as a ratio between raw and fitted values for each year (Fritts, 1976; Cook and Kairiūkštis, 1990). Finally, the mean chronologies per forest type were obtained by first removing the autocorrelation (“prewhitening” in dplR) and then applying a bi-weight robust mean (Cook and Kairiūkštis, 1990). To assess the correlation between climate and detrended wood anatomical traits at annual EW and LW resolution, the function “*dcc*” from the R package *treeclim* (Zang and Biondi, 2015) was used. Pearson’s correlation was applied to the interval 1958 –2019 for the Polish site and for the period 1990 –2018 for the Spanish one, according to the age of the trees and to the availability of climatic records. Confidence intervals and significance (*α*< 0.05) of the correlation were calculated by means of bootstrapping (Biondi and Waikul, 2004). The climatic variables considered for the analyses were mean monthly temperature, monthly precipitation, and SPEI, calculated at a 6-monthly mean window, because the best correlations were found at this resolution. For this analysis, the climatic variables were detrended identically to the tree-ring and wood anatomical series to remove the same amount of frequency from the series and avoid distortion in correlation coefficients (Ols et al., 2023).

#### 2.4.2 Intra-annual resolution analyses to detect drought effects between forest types and sites

To assess possible differences in intra-annual profiles of xylem traits between the two forest types, median values for each sector per tree were normalized by means of the minimum–maximum technique to account for individual tree variability and for the axial widening of xylem traits. Therefore, the minimum value of the wood anatomical trait profile of a given ring (x) gets converted to 0, whereas the maximum value gets converted to 1. Every remaining value gets converted into a decimal between 0 and 1 by means of the following formula:


$$\;x=\;\frac{x-min\left(x\right)}{\max\left(x\right)-min\left(x\right)}$$

An intra-annual profile per forest type, spanning the whole chronology, was obtained (Castagneri et al., 2020) by averaging the normalized values of each tree. Each intra-annual profile was smoothed with a loess function to reduce high-frequency variability and plotted with its confidence interval set at 95%. The non-parametric Komolgorov–Smirnov test was used to detect differences in the distribution of intra-annual xylem traits during drought between pines growing in pure and mixed forests in both sites. Drought years were identified by SPEI, calculated at 3-month resolution, starting from March to October, when the index started showing values below −1.5. In addition, intra-annual profiles of wood anatomical traits during drought and during non-drought were obtained by averaging the intra-annual drought year profiles and the non-drought ones. The Gini coefficient was computed to assess the degree of inequality of intra-annual xylem trait profiles during drought and non-drought periods. The Gini coefficient measures the inequality of a distribution varying between 0 to 1, where 0 represents perfect congruence and 1 represents the perfect incongruence (Biondi and Qeadan, 2008). Confidence intervals were calculated using the percentile, bias-corrected, and accelerated bootstrapping procedures.

Finally, to detect possible legacy effects on xylem traits related to drought events, intra-annual xylem trait profiles per forest type were obtained before, during, and after the year affected by drought. These profiles were obtained by averaging the intra-annual wood anatomical trait profiles 1 year before drought occurrence, the drought year, and the year after the drought event. Loess fit intra-annual profiles of wood anatomical traits were plotted 1 year before, during, and 1 year after the drought. Possible significant differences between xylem trait profiles of pines growing in pure and mixed forests at both sites were evaluated by applying the Kolmogorov–Smirnov test.

## 3 Results

### 3.1 Sensitivity of EW-LW anatomical traits to climate

**Figure 5:**
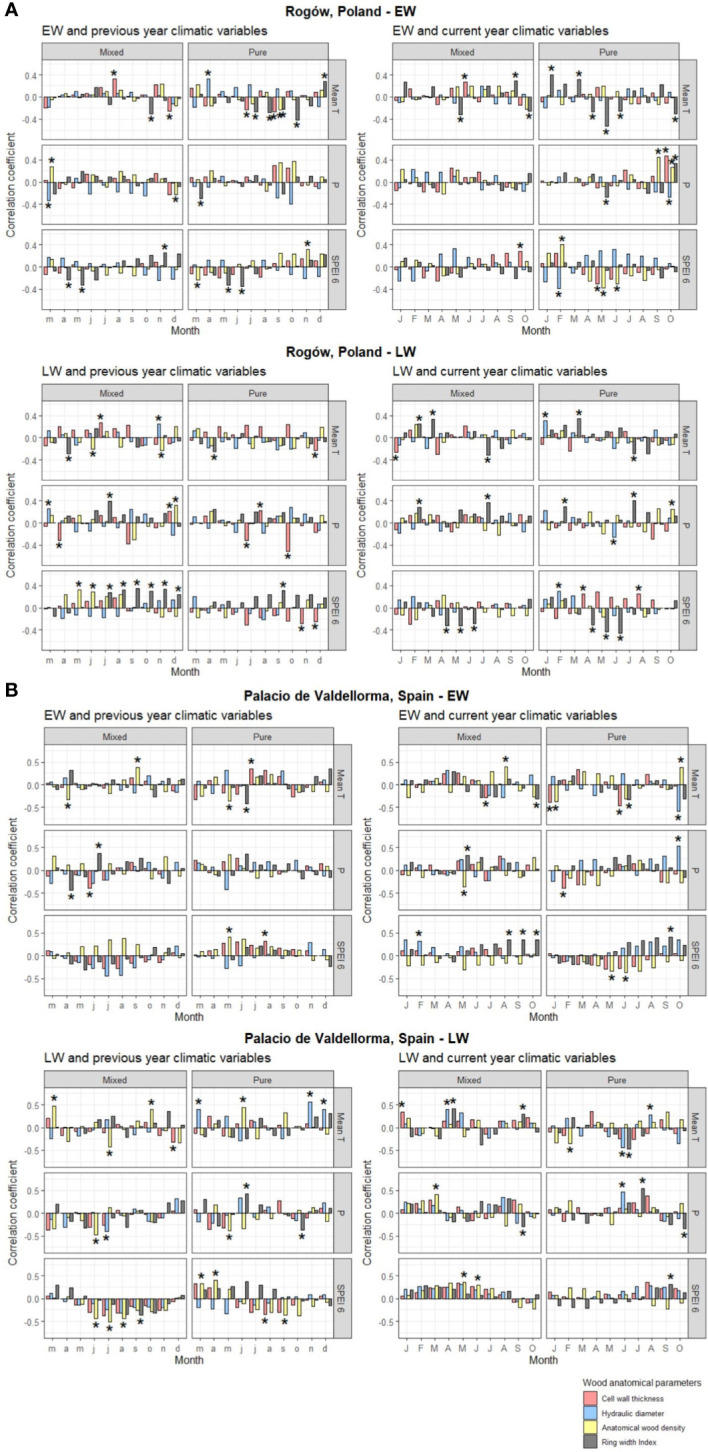
Pearson’s correlation for pines growing in Rogòw, Poland **(A)**, and Palacio de Valdellorma, Spain **(B)**, between monthly mean temperature, monthly precipitation, and Standardized Precipitation Evapotranspiration Index calculated at 6-month resolution (SPEI averaged within the previous 6 months starting from the month considered) of the previous year March to December (left) and current year January to October (right), and indexed chronologies of ring width, mean hydraulic diameter, cell wall thickness, and anatomical wood density of *P. sylvestris* in pure and mixed forest. The top panel shows correlations with EW traits and bottom panel with LW traits. Correlations were performed with the *dcc* function from “treeclim” R package, where the coefficients are univariate estimates of Pearson’s product moment correlation. The height of the bars represents the correlation coefficient, the bootstrap method applied provides confidence intervals, and the significance of the correlation is indicated by asterisks when p< 0.05. Time span considered for the analysis for the pines at the Polish site: 1958 –2018; for pines at the Spanish site: 1990– 2018.

The descriptive statistics of non-detrended wood anatomical traits in pure and mixed *P. sylvestris* stands are summarized in **Table 3**. In the correlation analyses, AD showed the highest responsiveness and was the best discriminant between forest types and sites. In the Polish site, temperature and SPEI strongly influenced EW traits, whereas LW traits also showed correlations with precipitation. In the Spanish site, all climatic variables were correlated with xylem traits (**Figures 5A, B**; for more statistical results, see **Table S1 in Supplementary Materials**).

**Table 3:** Descriptive statistics of raw wood anatomical traits grouped by site, forest type, and earlywood/latewood.

Site	Rogów				Palacio de Valdellorma			
Forest types	Mixed		Pure		Mixed		Pure	
EW-LW	EW	LW	EW	LW	EW	LW	EW	LW
No. of cells	553,055	456,421	531,626	454,015	409,225	156,657	396,670	167,347
DH (μm)	25.6 ± 8.3	13 ± 5.3	26.1 ± 8.4	13.7 ± 5.2	22.7 ± 7.9	10.9 ± 5.2	23.5 ± 7.9	10.6 ± 5.3
CWT (μm)	3.19 ± 0.75	5.39 ± 1.08	3.15 ± 0.76	5.45 ± 1.05	3.31 ± 0.7	4.86 ± 1.18	3.50 ± 0.69	5.08 ± 1.08
AD	0.13 ± 0.46	0.45 ± 0.56	0.13 ± 0.46	0.47 ± 0.53	0.22 ± 0.45	0.46 ± 0.57	0.24 ± 0.44	0.48 ± 0.58

Values are presented with ± standard deviation. DH, mean hydraulic diameter; CWT, cell wall thickness; AD, anatomical wood density.

#### 3.1.1 Inter-annual responses of EW to climate

Pines in pure forest stands presented similar responses to a set of climatic variables in both sites indicating that, when droughts occurred in May to June, trees produced EW with higher AD (and increased carbon investment into CWT in Poland). EW traits of such pines showed stronger correlations with previous and current year’s temperatures (and current SPEI in the case of Poland) compared with pines growing in mixed stands (**Figures 5A, B**). In pure pine stands, previous May to July temperatures decreased EW width in both sites. They also reduced CWT and AD in trees growing in Polish (July) and Spanish (May) sites, respectively (**Figures 5A, B**).

#### 3.1.2 Inter-annual responses of LW to climate

Tree admixture minimally influenced LW traits, which showed similar responses between sites and forest types. As an example, CWT and AD negatively correlated with previous April and July precipitation (mixed and pure forest, respectively) in the Polish site and with previous June and May precipitation (mixed and pure forest, respectively) in the Spanish site (**Figures 5A, B**). LW traits also showed different responses in each site, independent of tree admixture. In Poland, at both forest types, current February/July precipitation and temperature increased LW width. Conversely, it was reduced by the previous April/current July temperature and by previous summer droughts (**Figure 5A**). In the Spanish site, in both forest types, AD in LW increased following previous summer droughts (in pure forest also CWT; **Figure 5B**).

#### 3.1.3 Site-dependent EW and LW responses to climate

The following different results in climate correlation analyses between forest types highlighted the role of tree admixture in pine response to local climate. In the Polish site, only in the pure pine stands, EW width increased with temperature in January and March, whereas higher April to May and June temperature reduced it (**Figure 5A**).

In the Spanish site, xylem trait–climate associations were less dependent on admixture and stand density. The EW CWT and AD negatively correlated with precipitation for pines growing in pure (January) and in mixed forest (May), respectively. In the latter, EW width increased with May precipitation. In both forest types, a rise in previous April/May temperature reduced EW AD, whereas higher temperature in July and September increased carbon investment into CWT (pure forest) and coincided with an increase in AD (mixed forest; **Figure 5B**). Previous rise in November/December temperature favored larger DH in LW in the pure pine forests and was related to a decrease in CWT in LW in pines in mixed forest. Previous summer droughts increased AD in LW in both forest types (also CWT for pure forest pine). AD was reduced in LW for pines in pure forest when drought occurred during previous March/April and similarly for pines in mixed forest, when drought occurred during current May/June (**Figure 5B**).

### 3.2 Analyses of intra-annual wood anatomical profiles

#### 3.2.1 Intra-annual responses of wood anatomical traits to drought

At the Polish site, severe drought events occurred in the following years: 1959, 1963, 1972, 1982, 1983, 1989, 1990, 1992, 2000, 2006, 2007, 2011, 2012, 2014, 2015, and 2018; at the Spanish site: 1991, 1993, 1995, 1997, 2000, 2001, 2005, 2010, 2012, 2015, 2016, 2017, and 2018 (cf. **Figure 2**). The intra-annual profiles of wood anatomical traits in pure and mixed forests partly overlapped for most of the years in both sites, showing no evident pattern linked to tree admixture (Polish and Spanish sites, **Figures S1A–C**; Poland and Spain, **Figures S2A–C**; **Supplementary Materials**). Trees grown at the Spanish site showed age differences; therefore, we plotted intra-annual profiles following their ontogenesis (**Figures S3A–C**). Pine profiles growing in the two forest types showed no difference. A Kolmogorov–Smirnov test applied to the years characterized by drought highlighted significant differences between pines growing in the two forest types but no consistent pattern among forest types and sites (**Figures S1A–C, S2A–C**).

**Figure 6:**
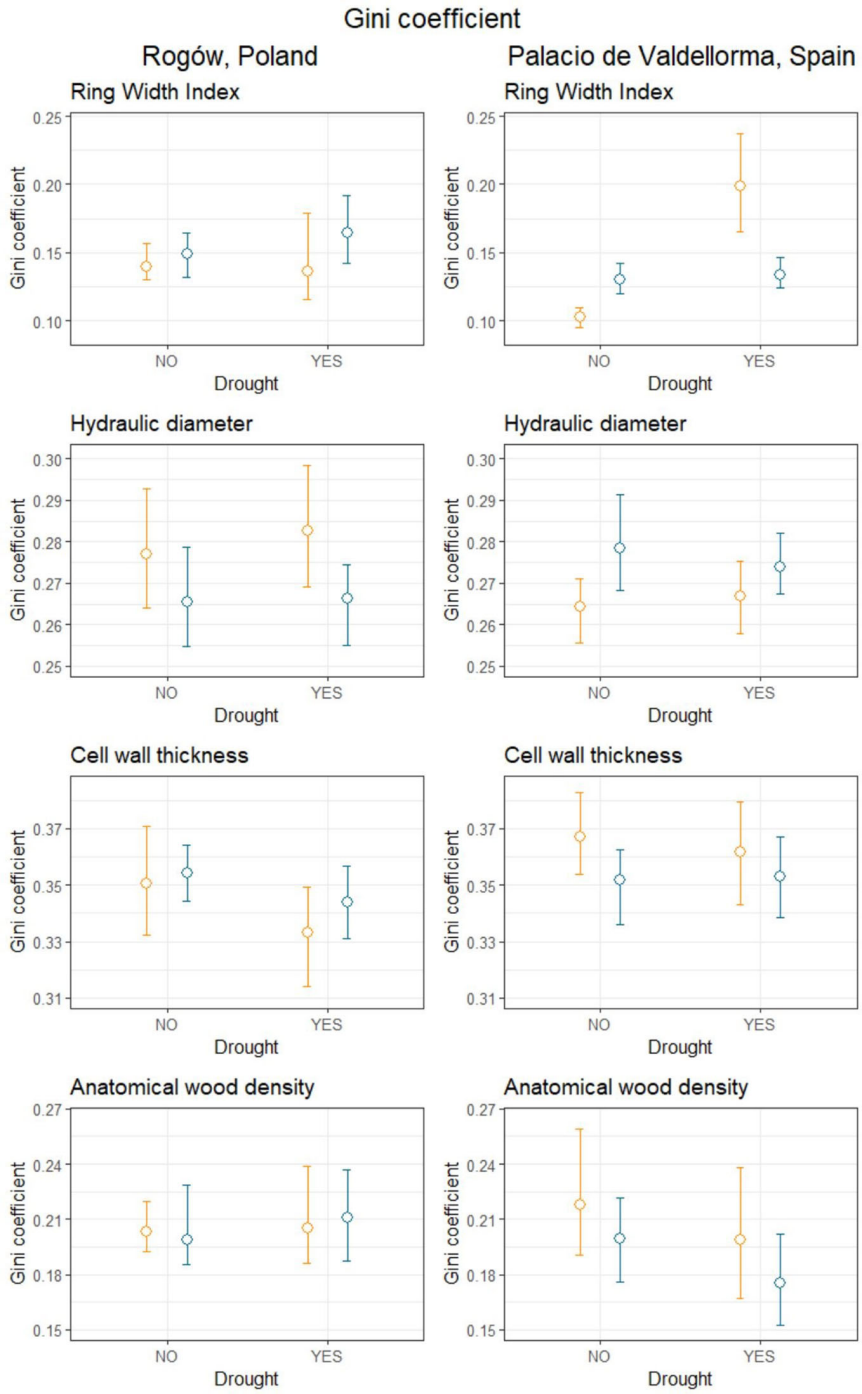
Gini coefficient calculated for mean hydraulic diameter, cell wall thickness, anatomical wood density and ring width index during years when drought occurred (YES) and during years characterized by absence of drought (NO), divided into mixed (orange) and pure (blue) forests, in Poland (left panel) and in Spain (right panel). Empty circles represent the mean Gini coefficient, and the upper and lower bound represent the bootstrapped 95% confidence intervals calculated using the percentile, bias-corrected and accelerated (BCa) method.

**Table 4** summarizes the Gini coefficients per intra-annual profiles aggregated by sites, forest types, and drought events. At the Polish site, the Gini coefficient did not reveal significant differences in variability between the profiles. At the Spanish site, the Gini coefficient did not highlight differences in wood anatomical traits except for ring width index (RWI). During the absence of drought, pines in mixed forests showed lower Gini coefficients compared with pines in pure forests. On the contrary, during drought, pines in pure forests showed lower Gini coefficients compared with pines in mixed forests (**Figure 6**).

**Table 4:** Gini coefficient calculated for mean hydraulic diameter (DH), cell wall thickness (CWT), anatomical wood density (AD), and ring width index (RWI) in mixed and pure forests during years with absence of drought (NO) and drought conditions (YES).

SITE	Rogów				Palacio de Valdellorma			
FOREST TYPE	Mixed		Pure		Mixed		Pure	
DROUGHT	NO	YES	NO	YES	NO	YES	NO	YES
DH upper CI	0.293	0.298	0.279	0.274	0.271	0.275	0.291	0.282
DH Mean Gini	0.277	0.283	0.266	0.266	0.264	0.267	0.278	0.274
DH lower CI	0.264	0.269	0.255	0.255	0.256	0.258	0.268	0.268
CWT upper CI	0.371	0.349	0.364	0.357	0.383	0.380	0.362	0.367
CWT Mean Gini	0.351	0.333	0.354	0.344	0.367	0.362	0.352	0.353
CWT lower CI	0.332	0.314	0.344	0.331	0.354	0.343	0.336	0.338
AD upper CI	0.220	0.239	0.229	0.237	0.259	0.238	0.221	0.202
AD Mean Gini	0.203	0.205	0.199	0.211	0.218	0.199	0.199	0.175
AD lower CI	0.192	0.186	0.186	0.188	0.191	0.167	0.176	0.152
RWI upper CI	0.157	0.179	0.165	0.192	**0.110**	**0.237**	**0.142**	**0.146**
RWI Mean Gini	0.140	0.136	0.149	0.165	**0.103**	**0.199**	**0.130**	**0.134**
RWI lower CI	0.130	0.116	0.132	0.143	**0.095**	**0.165**	**0.120**	**0.124**

Bootstrapped 95% confidence intervals **(CI)** were calculated using the percentile, bias-corrected and accelerated (BCa) procedure. Bold values highlight significant differences of Gini coefficients.

#### 3.2.2 Drought legacy effects on intra-annual wood anatomical traits

**Figure 7:**
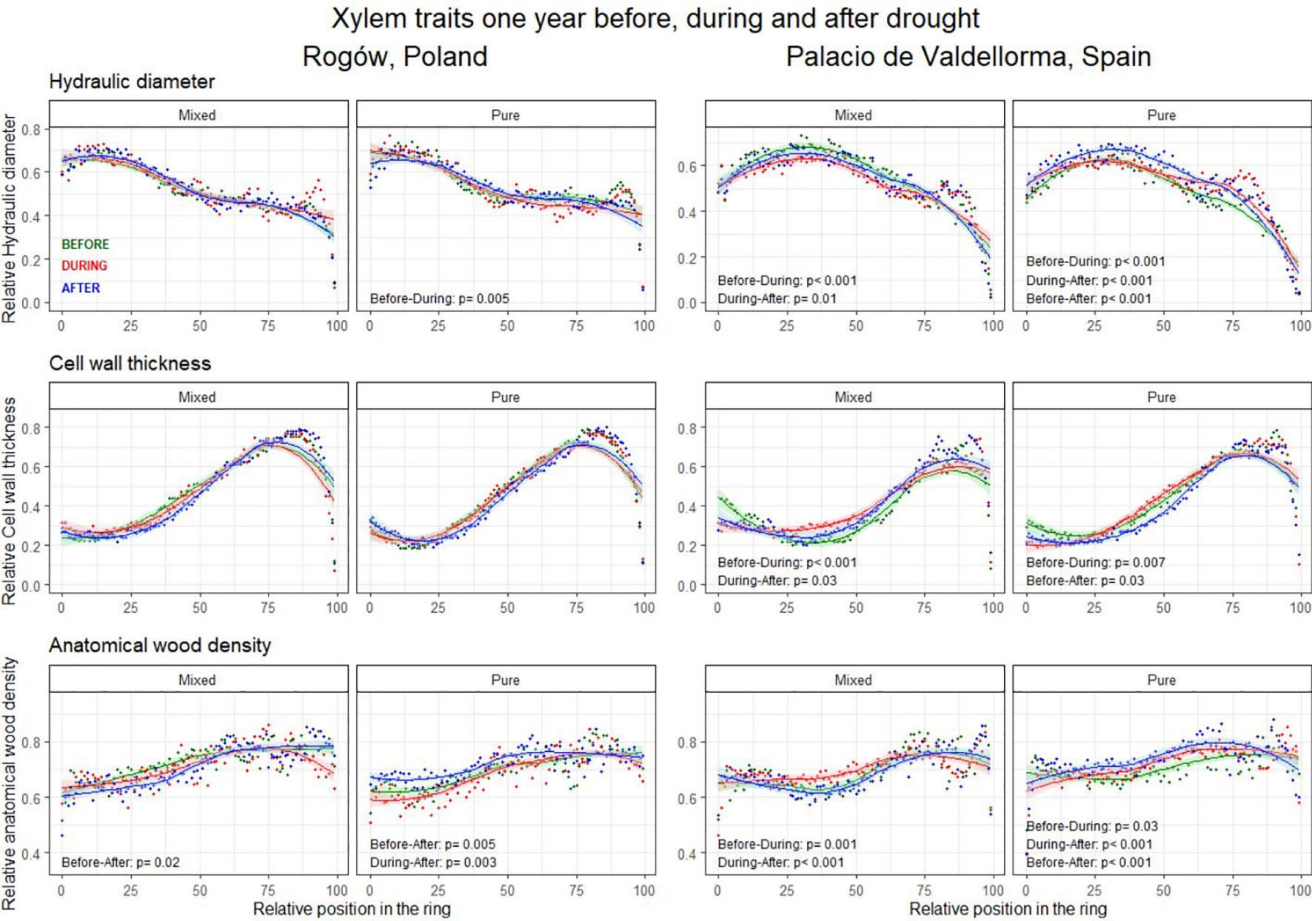
Intra-annual profiles of mean hydraulic diameter (top), cell wall thickness (middle), and anatomical wood density (bottom) aggregated per drought occurrences. The green lines represent the intra-annual profile 1 year before the drought, the red lines during the drought, and the blue lines 1 year after the drought. Possible differences between wood anatomical trait profiles are highlighted with the Kolmogorov–Smirnov test and presented when significant (p< 0.05).

The intra-annual analyses of xylem trait profiles conducted 1 year before, during, and 1 year after drought events revealed AD to be a fundamental trait showing contrasting legacy effects related to drought events for the two forest types (**Figure 7**; **Table S2** in **Supplementary Materials**).

In pure pine stands, on both sites, AD increased significantly 1 year after the drought compared with the pre-drought profile (Polish site, *p* = 0.005; Spanish site, *p*< 0.001) and to the during-drought profile (Polish site, *p* = 0.003; Spanish site, *p*< 0.001). Conversely, in mixed pine stands, AD decreased significantly 1 year after the drought compared with the pre-drought profile and to the year when drought occurred (*p* = 0.02 and *p*< 0.001, Polish and Spanish sites, respectively).

At the Polish pure pine stand, the DH profile decreased significantly during drought compared with the pre-drought profile (*p* = 0.005). CWT profiles did not present significant differences.

At the Spanish site, the DH profiles in the two forest types differed. In the mixed pine forest, they decreased significantly during drought compared to the previous year (*p*< 0.001). In the pure pine stand, they showed the opposite trend, although the increase was only significant in the last sectors of the ring. In pure pine stands, DH showed a further increase 1 year after the drought compared with drought and pre-drought conditions (*p*< 0.001). In both forest types, CWT profiles increased during drought compared with the previous year (*p*< 0.01). CWT decreased significantly 1 year after the drought compared with drought and pre-drought conditions (*p* = 0.03; in mixed and pure forests, respectively). In addition, AD increased during drought compared with the pre-drought profile in both forest types (pure forest, *p* = 0.001; mixed forest, *p* = 0.03; **Figure 7**).

## 4 Discussion

We aimed to understand whether tree admixture influences *P. sylvestris* responses to climatic variability and drought events, under contrasting climatic conditions, namely, Continental versus Mediterranean. Considering previous studies, ring width usually decreased during drought events, because the carbon sequestration is considerably reduced during drought (Bréda et al., 2006; Brunner et al., 2015), leading to the formation of narrow rings. On the basis of our results, ring width was not directly affected by the observed droughts, and it did not show a consistent decrease in response to these events (**Figure 4**). Under such conditions, xylem traits provide higher-sensitivity and higher-resolution information on ecophysiological responses to climatic variability and drought, a feature often lacking in the most common studies based on ring-width proxies (Carrer et al., 2014).

At both sites, mixed and pure forest stands were very close to each other. Consequently, elevation and exposition, soil composition, and texture are homogeneous factors that do not represent sources of variability influencing tree responses to climate. At the Polish site, tree age and degree of tree competitiveness (measured with the Stand Density index) were comparable between forest types, and we discarded them as sources of variability (**Tables 1**, **2**). At the Spanish site, pine trees in pure stands were older than in mixed stands (36 and 23 years old, respectively; **Table 2**) and were in the juvenile growth phase, which usually feature significantly different growth dynamics and climate responses than older trees (Carrer and Urbinati, 2004). Concerning xylem traits, they are known to be “size-dependent” rather than “age-dependent” (Anfodillo et al., 2013). In our study, tree height varies (**Table 2**), and standardization techniques that we applied are prerequisites to accurately interpret and compare xylem traits (Carrer et al., 2014). Regarding the Spanish site, the Stand Density Index was significantly higher in pure stands than in mixed stands (**Table 1**). In this case, a different level of competition between forest types did not produce a univocal correlation of the results to the tree admixture.

### 4.1 Similar wood anatomical trait responses to climate and drought at both sites

Our findings showed that, at both sites, EW traits correlated strongly with previous-year climatic conditions, especially temperature, in accordance with previous studies (Martin-Benito et al., 2013; Pellizzari et al., 2016; Björklund et al., 2017), likely due to the role of photoassimilates accumulated during the previous vegetative season (Kagawa et al., 2006; von Arx et al., 2017).

Overall, pines growing in pure forest showed a stronger correlation with temperature compared with pines in mixed stands, which, in turn, showed lower sensitivity to temperature variability (**Figures 5A, B**). This reduced sensitivity could suggest increased resistance of mixed-forest pines to climate variability (Chmura et al., 2011; Magruder et al., 2013; Thurm et al., 2016), which could be related to higher structural heterogeneity, increased canopy plasticity, and extended canopy occupation compared to monospecific stands (Pretzsch et al., 2015). Canopy cover is a key factor influencing forest microclimate by buffering low and high temperature extremes, and its effects are more pronounced during the vegetative season (Greiser et al., 2018). Only few studies considered the effects of different canopy cover on forest microclimate. Different temperatures were observed in pure pine versus pine-broadleaved stands (Porté et al., 2004; Perot et al., 2021). In the latter study, substantial differences were observed regarding tree phenology due to a different temperature perception experienced by trees in mixed forest.

Our results suggested that, in mixed forest, more stable microclimatic conditions occurred, and climatic resistance of trees improved, confirming our first hypothesis stating that *P. sylvestris* in mixed forest shows lower climate sensitivity. This indicates that mixed *P. sylvestris* forests are desirable in the face of climate change to buffer against the climatic extremes that are projected for the near future.

Despite the limited plasticity of EW traits in response to current climatic conditions, they are known to strongly depend on water availability, especially tracheid size (Bryukhanova and Fonti, 2013; Cabon et al., 2020). When drought occurs during spring, cavitation can impair EW functionality, which is the main tissue responsible for water transport (Martín et al., 2010). The sensitivity of *P. sylvestris* to spring drought was reported under both climatic conditions –Continental and Mediterranean– considered in this study (Camarero et al., 2011; Michelot et al., 2012). At both our sites, late-spring drought increased the current AD (in Polish site, also CWT) in EW in pure forest, whereas pines in mixed forest did not respond in these parameters (**Figures 5A, B**). Intra-annual wood anatomical trait analyses conducted to assess drought legacy effects on pines consistently revealed that pines growing in pure forest produced denser wood 1 year after the drought (**Figure 7**). These adjustments, performed by pines growing in pure forest, generated a safer wood with reduced cell implosion and thus cavitation risk (Hacke et al., 2001). They also indicated higher sensitivity to drought and legacy effects, which can remain visible for several years in wood anatomical traits (Pacheco et al., 2018a; Castagneri et al., 2020). Different competitive ability between trees growing in pure and mixed forests emerged during stressful events such as during spring drought (Paquette and Messier, 2011; Pretzsch et al., 2013b). This led to a harsher level of competition for pines growing in pure forest but ameliorated conditions for pines growing in mixed forest. Indeed, the latter presented a reduction in AD 1 year after the drought, showing no legacy effects (**Figure 7**). During drought periods, in mixed forests, contrasting water-use strategies displayed by the two tree species could have been improving the efficiency of water use. Complementarity in root plasticity and distribution could increase nutrient availability and uptake (Pretzsch et al., 2013b; Bello et al., 2019). Canopy interaction and complementarity are thought to produce lower competition for light (Pretzsch et al., 2020) and alter microclimatic conditions by shading and by reducing evapotranspiration (van Loon et al., 2014; Hatfield and Dold, 2019); these processes could be improving conditions for pines in mixed stands. In those pines, carbon resources could be allocated in other tree compartments, i.e., the canopies (Merlin et al., 2015), promoting needle elongation and primary growth, likely increasing tree competitiveness (Matisons et al., 2019). Such processes occur during late spring and could be impeded or slowed down by spring drought (Richardson, 2000).

At the Spanish site, the pure forest stands were denser than the mixed forest stands. Higher density could imply that pure pine stands could experience a higher level of competition exacerbated by drought stress. Indeed, our results showed higher sensitivity to drought of pines growing in pure stands compared with mixed stands. This makes it difficult to interpret the results univocally, and, although they would be in line with our expectation, the higher sensitivity to drought seen in pines growing in pure stands at the Spanish site cannot be attributed only to tree admixture as a single cause but also to stand density. Hence, our first hypothesis stating that *P. sylvestris* growing in pure stands will present higher xylem modifications in response to drought events compared with *P. sylvestris* growing in admixtures with *Quercus* sp. can only be partially confirmed.

LW traits showed similar correlations with climatic variables at both sites and in both forest types. Pine responded to previous spring and summer precipitation without allocating carbon resources to reinforce the hydraulic system, showing a decrease in CWT and in AD. This is in line with previous studies (Björklund et al., 2017; Știrbu et al., 2022), and it indicates the presence of a time lag in the influence that previous-year climatic conditions have on LW traits, despite their high responsiveness to current climatic conditions (Eilmann et al., 2006; Seo et al., 2008; Martin-Benito et al., 2013; Cuny and Rathgeber, 2016). These results also showed that *P. sylvestris* LW traits respond in similar ways to spring and to summer precipitation, thereby presenting precise regulatory mechanisms, which likely integrate local genetic adaptation and phenotypic plasticity into a coordinated response to climate; despite the different provenances of the trees, the local climate, and the different stand density at the Spanish site.

Correlation with climatic variables, coupled with the trend of the intra-annual wood anatomical profiles, where most changes occurred in the first two/third sectors of the ring, indicated a negligible effect of tree admixture when droughts occurred during the late growing season. This indicates that the benefits of inter-specific facilitation mechanisms become relevant during the first part of the growing season and are mainly visible in EW traits and during stressful events, such as spring drought. This inference is coherent with the stress gradient hypothesis stating that facilitation mechanisms could be more evident under harsher conditions such as under resource limitation (Callaway and Walker, 1958; Paquette and Messier, 2011; Pretzsch et al., 2013b). Our findings suggested that inter-specific facilitation mechanisms are likely influenced by the phenology of the species considered (Merlin et al., 2015), a factor that was neglected in the most studies that investigated the effect of tree admixture on tree performance (Del Río and Sterba, 2009; Pretzsch et al., 2013a; Bielak et al., 2014; Pretzsch et al., 2015; Thurm et al., 2016; Zeller et al., 2017; Pretzsch et al., 2020).

### 4.2 Site-dependent wood anatomical trait response to climate variability and drought

Cell enlargement tends to vary significantly with species and with site conditions and is highly sensitive to water stress (Cabon et al., 2020). At the Polish site, small variations of DH were reported (**Figures 5A**, **7**). This suggested that the site was not water-limited and that *P. sylvestris* wood anatomical traits were mainly under the influence of temperature, as assessed by several studies on conifer species in temperate climate (Seo et al., 2008; Liang et al., 2013; Björklund et al., 2017). Temperature influences many physiological processes, from photosynthate availability to wall thickening (Cuny and Rathgeber, 2016) for which it is considered a fundamental driver (Körner, 2003; Cuny et al., 2015). In Central Poland, high winter/late-winter temperatures positively influence *P. sylvestris* growth, lengthening the growing season (Koprowski, 2012; Misi and Náfrádi, 2016; Misi et al., 2019; Giberti et al., 2022). In our study, we found interesting local differences driven by the influence of tree admixture on xylem traits. Only pure-stand *P. sylvestris* EW width correlated positively with temperatures in January and March. On the contrary, pines growing in mixed stands did not show such correlations (**Figure 5A**). In mixed forests, during winter and late winter, we expect slightly higher air temperature: given the absence of oak leaves, the sunlight penetrates more efficiently in comparison with closed canopies of the pure *P. sylvestris* forests. Increase in winter temperatures presumably provided *P. sylvestris* in the pure stands with an amount of energy sufficient to anticipate and lengthen the growing season. Conversely, LW width presented similar correlations in both forest types. It was positively correlated with precipitation in spring and summer, and negatively correlated with current temperature and drought, in line with previous studies (Liang et al., 2013; Martin-Benito et al., 2013; Matisons et al., 2019), re-confirming the negligible effect of tree admixture on LW traits.

At the Spanish site, xylem traits responded similarly to the climatic variables for both forest types, despite the different tree admixture and the different stand density (**Figure 5B**). Under a Mediterranean climate, water availability in summer is a primary tree growth limiting factor (Bogino et al., 2009; Martin-Benito et al., 2013; Cuny et al., 2015), and higher sensitivity is expected toward increased summer temperatures that can eventually become associated to seasonal drought. This is also what we observed in our case, where the previous summer temperature increased carbon investment into CWT (pure forest), and increased AD (mixed forest) in EW. Carbon investment into CWT was reduced following precipitation, showing adjustments that reduced hydraulic safety (Hacke and Sperry, 2001; Hacke et al., 2001; **Figure 5B**). Analyses of wood anatomical trait profiles of pines for both forest types showed that AD and CWT increased during drought, as observed in previous studies (Hacke and Sperry, 2001; Hacke et al., 2001; Desoto et al., 2011; Hereş et al., 2014; Pellizzari et al., 2016, **Figure 7**), probably improving the safety of the hydraulic system against cavitation risks. Understandably, pines in mixed forests also showed a smaller DH during drought events, whereas pure pine forests showed an increase in DH during drought. The maximization of hydraulic efficiency during drought, at the risk of cavitation, was also observed in pines in previous studies (Eilmann et al., 2009; Eilmann et al., 2011; Martin-Benito et al., 2013; Martin-Benito et al., 2017; Petrucco et al., 2017; Candel-Pérez et al., 2018; Guérin et al., 2020; Buras et al., 2023). However, a closer inspection of the intra-annual DH profile revealed that this trait increased only in the last portion of the ring (**Figure 7**). *Pinus sylvestris* growing under Mediterranean and Sub-Mediterranean climates shows secondary growth extended until October (Camarero et al., 2011; Prislan et al., 2016). In case of summer drought, a larger lumen and prolonged cambial activity could supply *P. sylvestris* hydraulic efficiency at the end of summer or early autumn, when better conditions in terms of temperature and water availability occur.

### 4.3 Intra-annual wood anatomical trait responses to drought at both sites

According to our second hypothesis, we expected harsher conditions in terms of drought at the Spanish site, which is located under a Mediterranean climate at the southern margin of *P. sylvestris*’ range. Consequently, we expected higher xylem trait variability for *P. sylvestris* trees, given the recurring drought that they are subject to. We could not confirm this hypothesis because we did not find evidence of higher xylem trait variability in pines in Spain (**Table 4**; **Figure 6**). In addition, we did not find consistent patterns at both sites suggesting that drought induces higher variability in xylem traits. Significant changes were reported at the Spanish site, where trees growing in mixed forests showed less constant growth rates (RWI) during drought than pines in pure forest, whereas the opposite occurred in the absence of drought; no differences were reported regarding other traits. Higher xylem trait variability was found in previous studies in dying or dead trees, in comparison with surviving ones after drought stress (Levanič et al., 2011; Hereş et al., 2014). During our study, we selected healthy and dominant trees to reduce the variability related to abiotic and biotic conditions; we chose sites with no evident limiting factors trying to isolate the sole effects of climate and tree admixture on pines.

Similar responses to climate were observed in pure pine xylem traits to both our sites. The influence of tree admixture was prominent in pines growing at the Polish rather than the Spanish site. The sampling strategy conducted at the Polish site allowed us to attribute xylem trait change between forest types to tree admixture as the sole factor, whereas, for the Spanish site, stand density could also play a role. Despite these sources of variability, pines growing at the Spanish site showed similarities in climate and drought responses, which could eventually indicate limited phenotypic plasticity. In the work of Richter et al. (2012), the Continental and Mediterranean populations of *P. sylvestris* were subject to water deficit in a common garden experiment. The authors reported reduced phenotypic plasticity in the southern *P. sylvestris* populations and hypothesized that southern provenance showed limited plasticity as a strategy to cope with the stressful environment where water deficit is recurrent. In our case, xylem trait adjustment was of modest magnitude at both sites, suggesting moderate or non-stressed conditions perceived by the trees (Martin-Benito et al., 2013).

Signs of tree mortality can be visible in xylem traits far in advance of the actual death (Pellizzari et al., 2016). Long-term risk of dieback following multiple single drought events may occur with the increase in severity and frequency of droughts (Petrucco et al., 2017; Dai et al., 2018). In this context, our findings provide interesting insights on xylem trait adjustments toward climatic variability and drought and the role that tree admixture plays in this context. For future studies, we recommend including suppressed and dying trees after drought stress along different stand density levels, to fully understand the role of tree admixture in modulating xylem traits and to investigate possible changes in inter-specific facilitation effects.

## 5 Conclusion

This study was carried out to understand whether tree admixture can influence wood anatomical trait responses to climatic variability and drought events. Among the wood anatomical traits considered, AD, being the synthesis of the cell wall and the lumen area, was one of the most sensitive traits to detect climate variability and drought responses under different climatic conditions and forest types. This corroborates the relevance of this trait as a proxy in the context of dendro-anatomical studies (Castagneri et al., 2020).

We showed that tree admixture significantly influenced xylem responses to climatic variables, and exerted legacy effects detectable during the year following drought events. Our findings highlighted that inter-specific facilitation mechanisms that occurred in the admixture between *P. sylvestris* and *Quercus* sp. become relevant in the first part of the growing season, during stressful events such as spring droughts and detectable in EW traits. This was evident considering the Polish site, although further investigations are needed for the Spanish site to disentangle the role of tree admixture from the one of stand density. In contrast, LW traits showed similarities in both forest types and sites, and this suggested negligible effects of tree admixture during the late growing season. On the basis of our findings, we suggest that inter-specific facilitation mechanisms are likely modulated by the phenology that characterizes the species considered.

A closer inspection of the intra-annual variability of xylem traits showed the potential role that tree admixture has in modulating xylem traits response in the face of climatic variability and drought events. This gains importance when considering that signs of tree mortality in xylem traits can occur far in advance of the actual death of trees (Pellizzari et al., 2016). Future climatic projections foresee a northward migration of *P. sylvestris*, and its decline in Central Europe by the end of the century (Buras and Menzel, 2019). Our results suggested that *P. sylvestris* could be subject to harsher competition in monospecific stands than that in mixed stands with *Quercus* sp., and this is in agreement with the findings of previous studies. Under current climatic conditions, this admixture could be adopted as a solution for an adaptive management strategy to increase the resilience of *P. sylvestris* to drought events.

## Data availability statement

The original contributions presented in the study are included in the article/**Supplementary Material**. Further inquiries can be directed to the corresponding authors.

## Author contributions

GT and CW initiated the collaboration project. CW developed the research idea for this study. GT, CW, KB, and FB planned the sampling design and the research activity. KB and FB were responsible for the dendrometric measurements of the experimental sites in Poland and Spain. GSG, GT, CW, KB, and BdT performed sample campaigns. EU was responsible for the first dendrochronological analyses of the cores with LIGNOSTATION™. GSG produced the wood anatomical samples. GSG, MC, and LU worked on the anatomical image acquisition from samples. The quantitative wood anatomical analyses were performed by GSG supervised by GA, AG, CW, and BT. GSG wrote the first draft of the manuscript and elaborated tables and figures with the help of AG, CW, KB, BT, and GA. CW supervised the study. All authors contributed to the revision of the manuscript and approved the final version.
