# Supplementary material for: The admixture of Quercus sp. in Pinus sylvestris stands influences wood anatomical trait responses to climatic variability and drought events
Source: Front Plant Sci. 2023 Nov 16;14:1213814. doi: 10.3389/fpls.2023.1213814 (PMC10687546; doi:10.3389/fpls.2023.1213814)
Supplement: Supplementary file 1 [file DataSheet_1.pdf]

Supplementary Material

The admixture of *Quercus sp.* in *Pinus sylvestris* stands influences wood anatomical trait responses to climatic variability and drought events

Giulia Silvia Giberti*, Georg von Arx, Alessio Giovannelli, Ben du Toit, Lucrezia Unterholzner, Kamil Bielak, Marco Carrer, Enno Uhl, Felipe Bravo, Giustino Tonon, Camilla Wellstein*

*** Correspondence:** giuliasilvia.giberti@unibz.it; camilla.wellstein@unibz.it

1 Supplementary Figures and Tables

1.1 Supplementary Figures

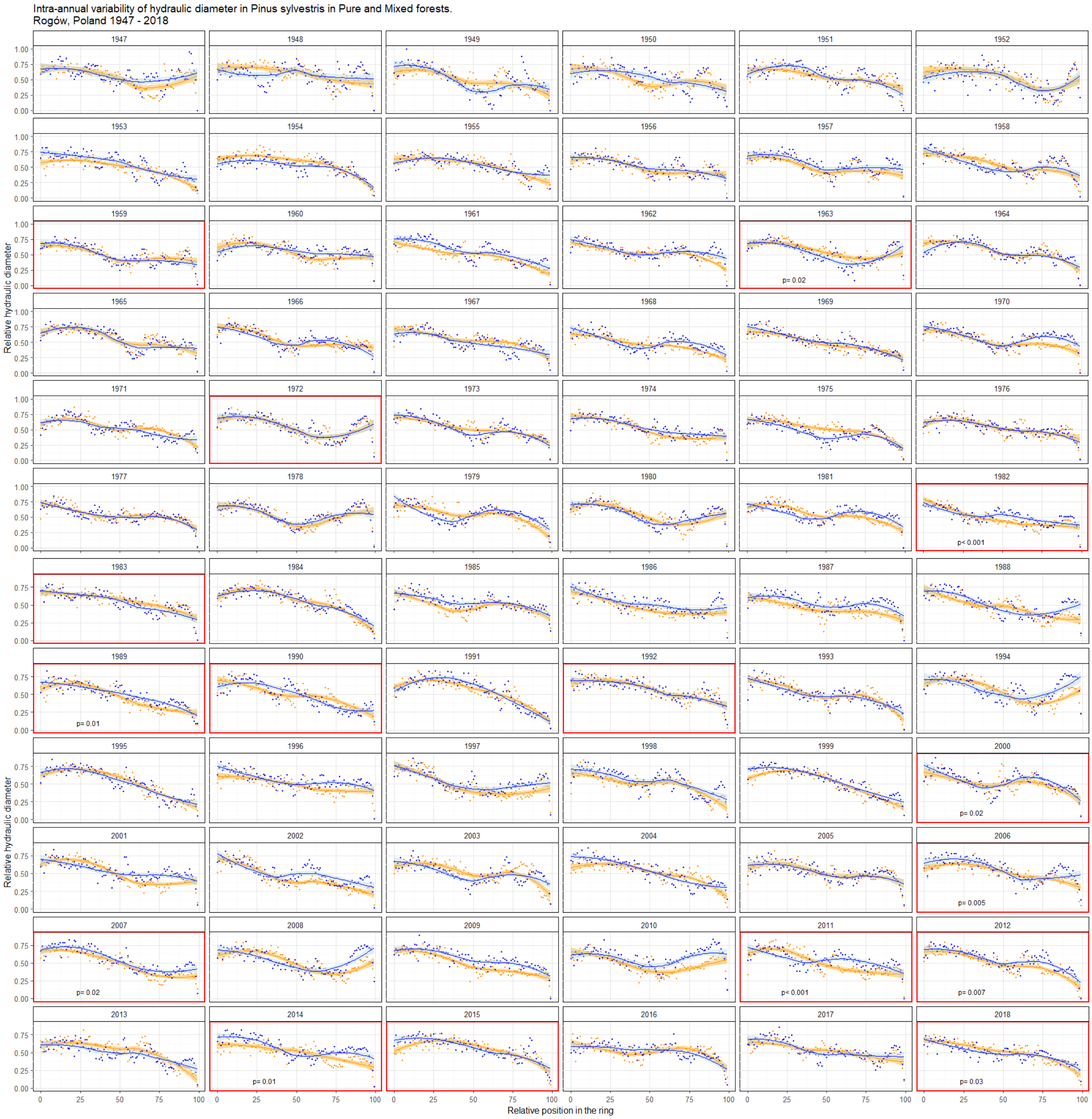

Supplementary Figure S1A Intra-annual profiles of mean hydraulic diameter aggregated per forest type plotted along the growing season of each year for the time span1945 – 2018. The intra-annual profiles are loess fit, the shaded area represents confidence interval set at 95%. Possible differences between wood anatomical trait profiles are highlighted with the Kolmogorov-Smirnov test and presented when significant ($p < 0.05$). Blue represents *P. sylvestris* in pure forest and orange *P. sylvestris* in mixed forest. Boxes marked in red indicate years when drought event occurred. Rogòw, Poland.

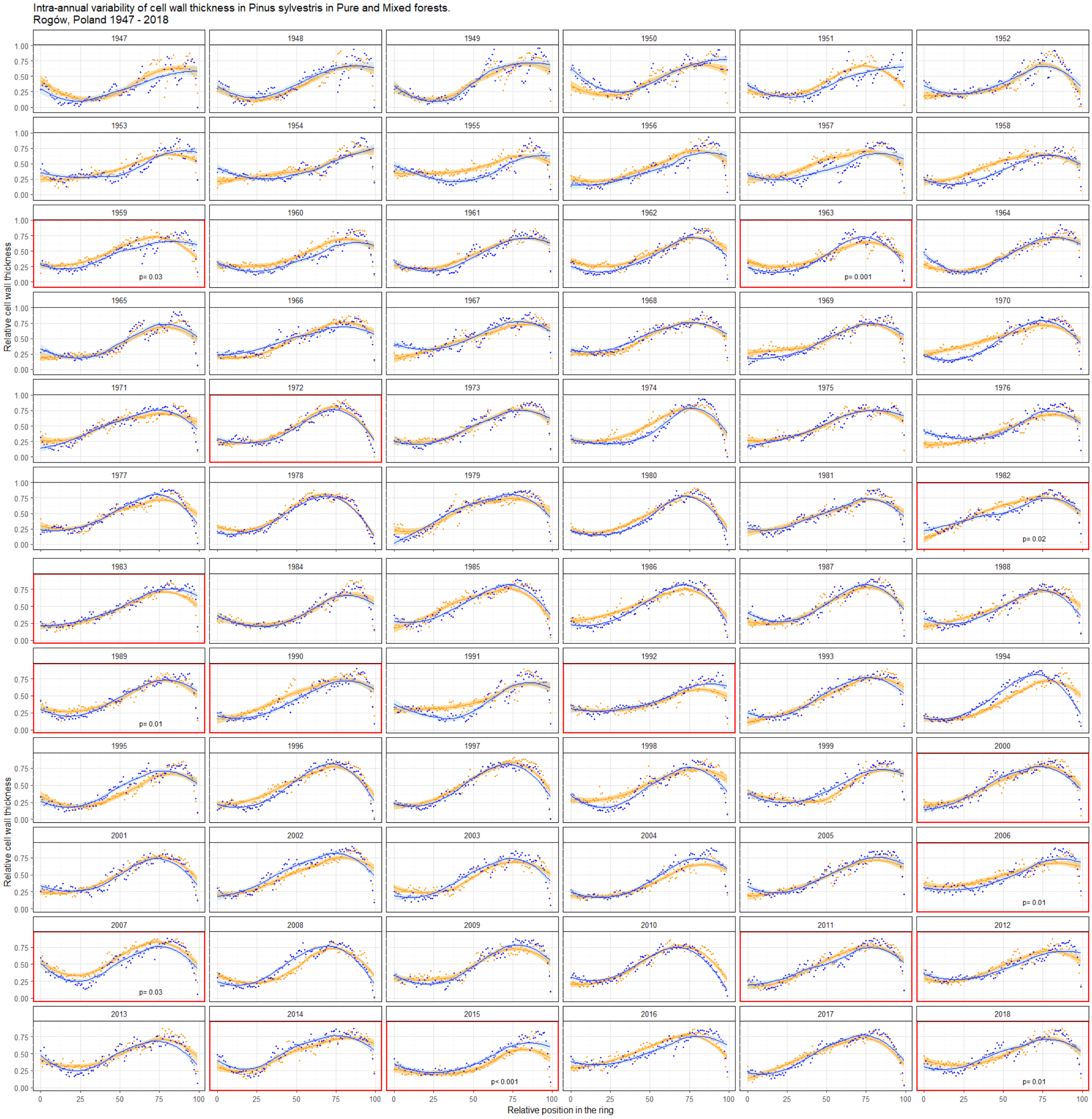

Supplementary Figure S1B Intra-annual profiles of cell wall thickness aggregated per forest type plotted along the growing season of each year for the time span 1945 – 2018. The intra-annual profiles are loess fit, the shaded area represents confidence interval set at 95%. Possible differences between wood anatomical trait profiles are highlighted with the Kolmogorov-Smirnov test and presented when significant ($p < 0.05$). Blue represents *P. sylvestris* in pure forest and orange *P. sylvestris* in mixed forest. Boxes marked in red indicate years when drought event occurred. Rogòw, Poland.

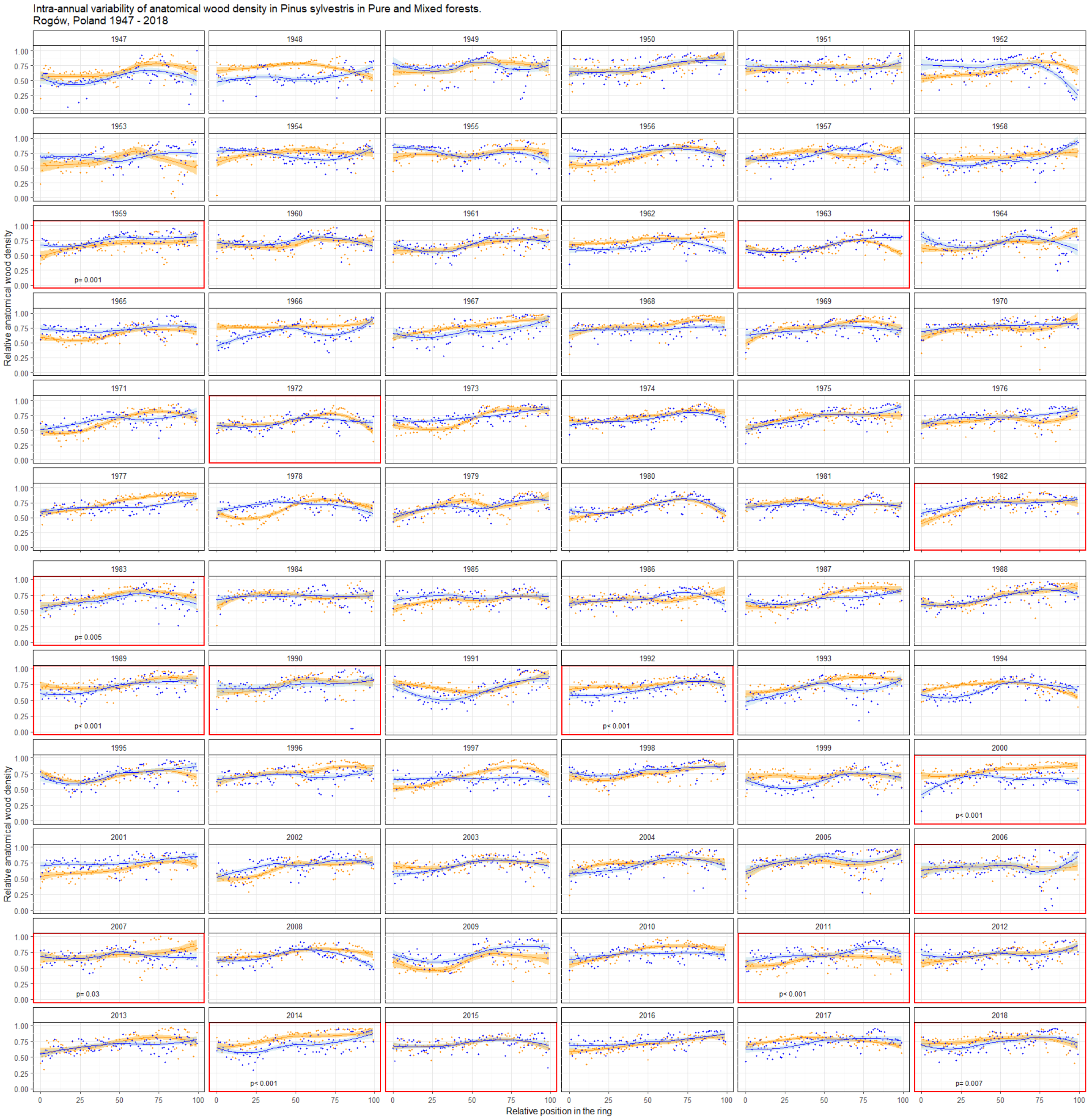

Supplementary Figure S1C Intra-annual profiles of anatomical wood density aggregated per forest type plotted along the growing season of each year for the time span 1945 – 2018. The intra-annual profiles are loess fit, the shaded area represents confidence interval set at 95%. Possible differences between wood anatomical trait profiles are highlighted with the Kolmogorov-Smirnov test and presented when significant ($p < 0.05$). Blue represents *P. sylvestris* in pure forest and orange *P. sylvestris* in mixed forest. Boxes marked in red indicate years when drought event occurred. Rogòw, Poland.

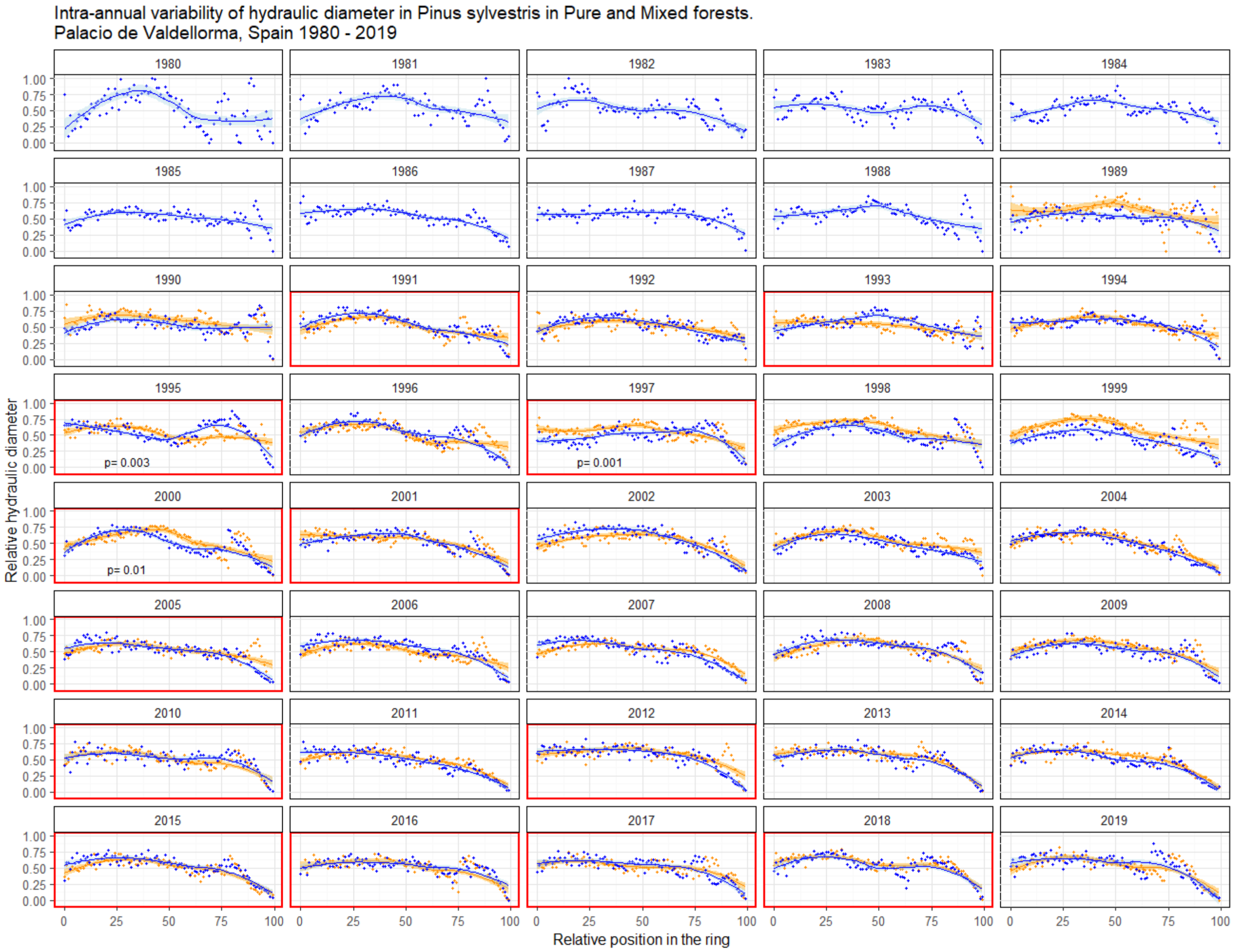

Supplementary Figure S2A Intra-annual profiles of mean hydraulic diameter aggregated per forest type plotted along the growing season of each year for the time span1980 – 2018. The intra-annual profiles are loess fit, the shaded area represents confidence interval set at 95%. Possible differences between wood anatomical trait profiles are highlighted with the Kolmogorov-Smirnov test and presented when significant ($p < 0.05$). Blue represents *P. sylvestris* in pure forest and orange *P. sylvestris* in mixed forest. Boxes marked in red indicate years when drought event occurred. Palacio de Valdellorma, Spain.

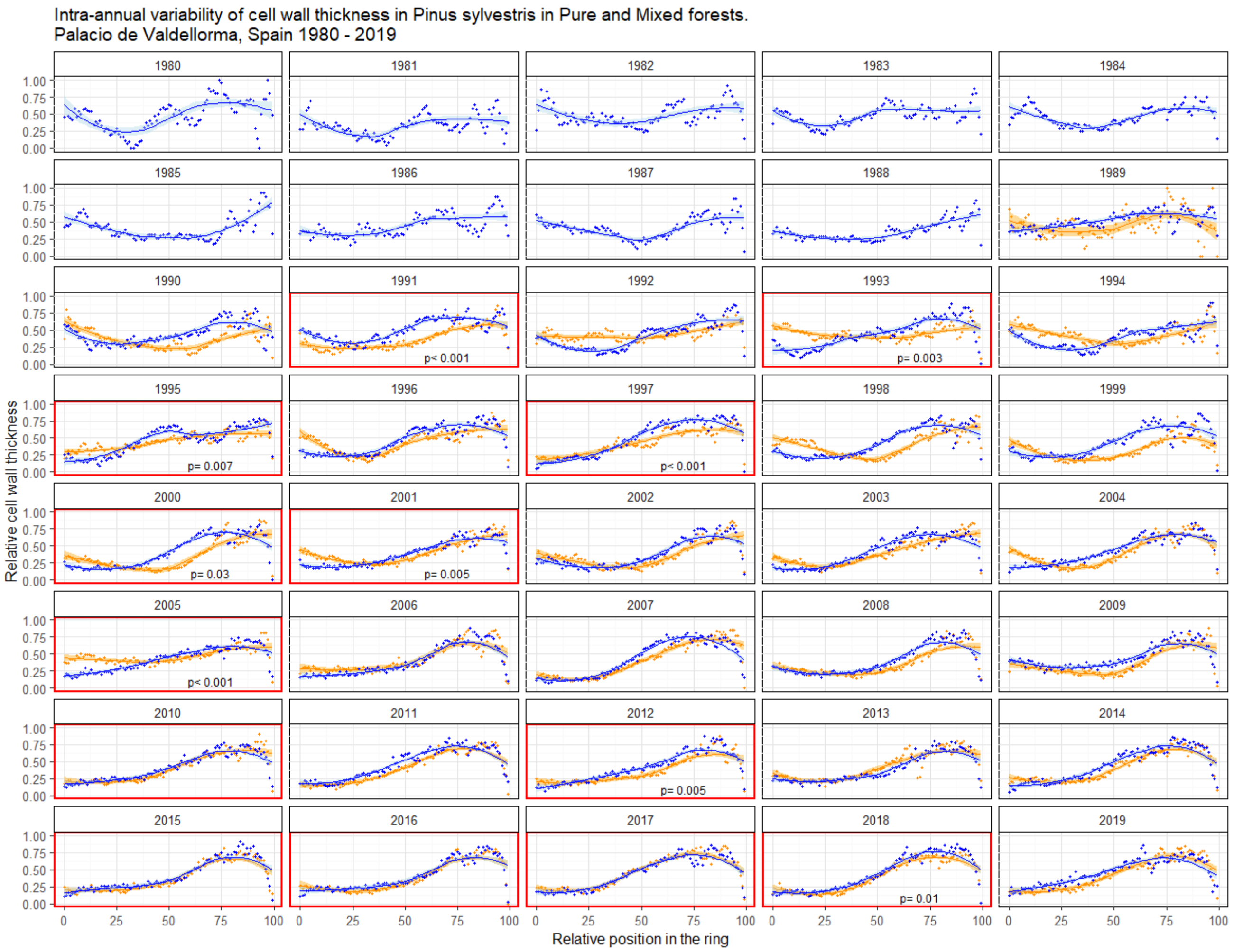

Supplementary Figure S2B Intra-annual profiles of cell wall thickness aggregated per forest type plotted along the growing season of each year for the time span 1980 – 2018. The intra-annual profiles are loess fit, the shaded area represents confidence interval set at 95%. Possible differences between wood anatomical trait profiles are highlighted with the Kolmogorov-Smirnov test and presented when significant ($p < 0.05$). Blue represents *P. sylvestris* in pure forest and orange *P. sylvestris* in mixed forest. Boxes marked in red indicate years when drought event occurred. Palacio de Valdellorma, Spain.

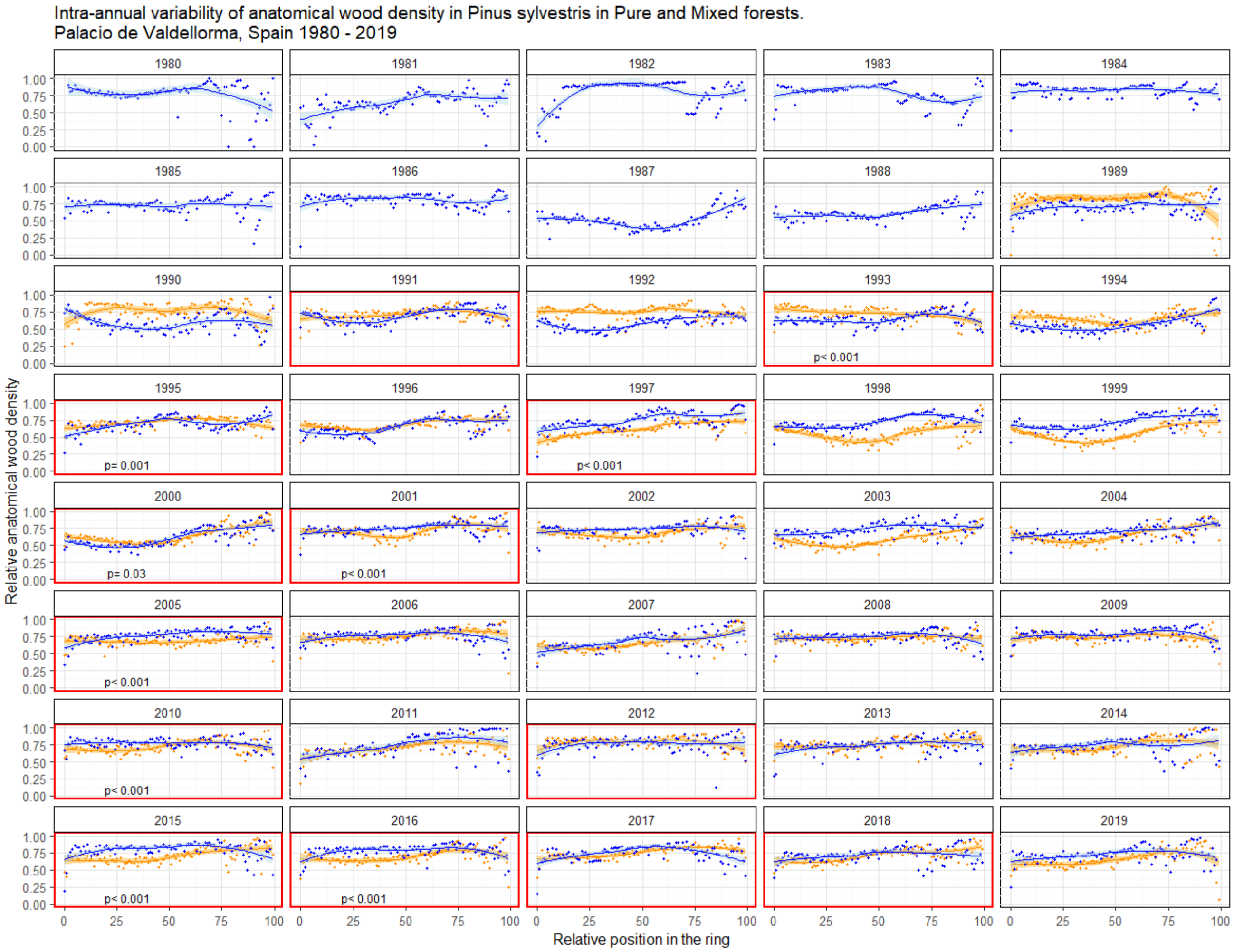

Supplementary Figure S2C Intra-annual profiles of anatomical wood density aggregated per forest type plotted along the growing season of each year for the time span 1980 – 2018. The intra-annual profiles are loess fit, the shaded area represents confidence interval set at 95%. Possible differences between wood anatomical trait profiles are highlighted with the Kolmogorov-Smirnov test and presented when significant ($p < 0.05$). Blue represents *P. sylvestris* in pure forest and orange *P. sylvestris* in mixed forest. Boxes marked in red indicate years when drought event occurred. Palacio de Valdellorma, Spain.

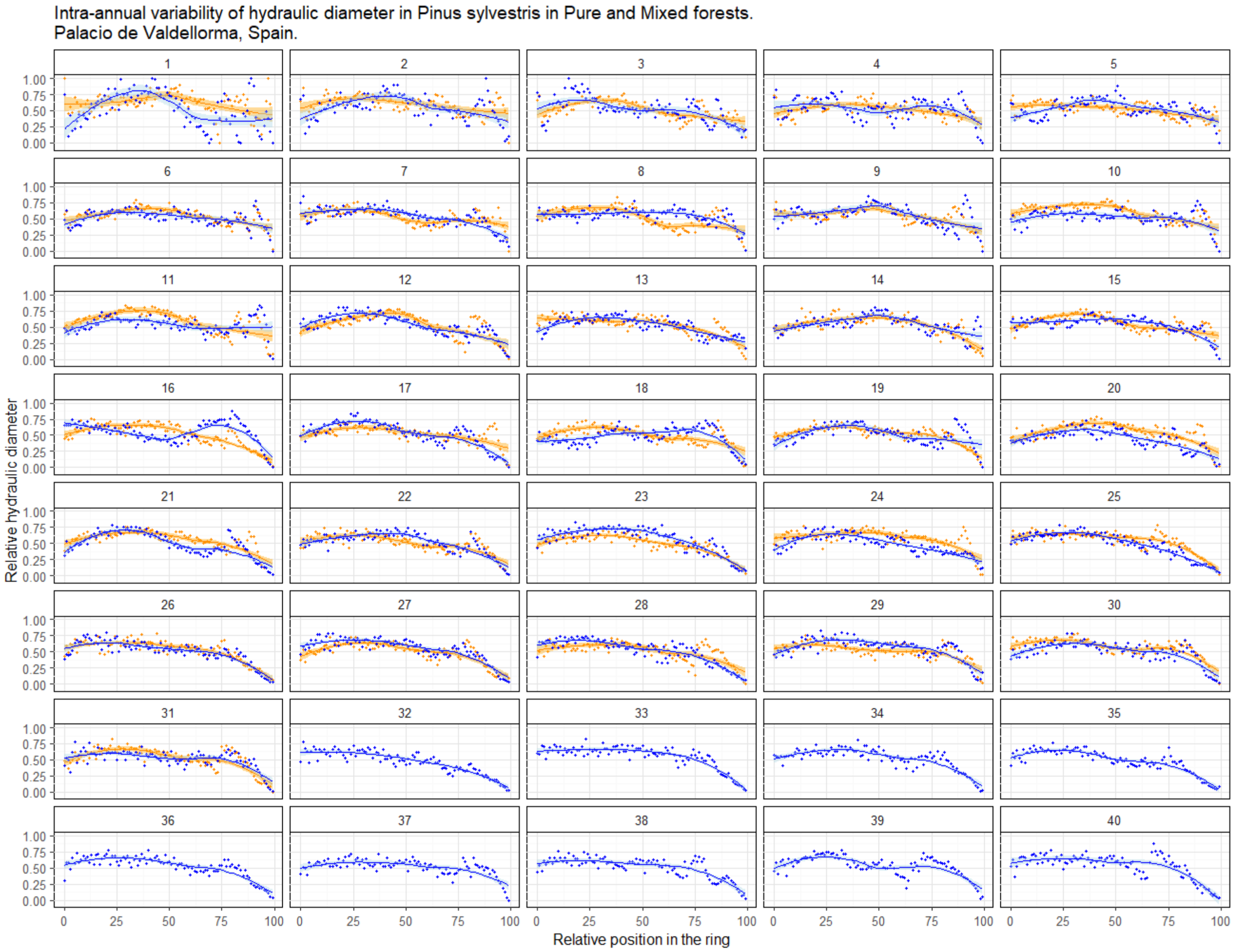

Supplementary Figure S3A Intra-annual profiles of mean hydraulic diameter aggregated per forest type plotted along the growing season of each year following the ontogenetic development of trees. The intra-annual profiles are loess fit, the shaded area represents confidence interval set at 95%. Blue represents *P. sylvestris* in pure forest and orange *P. sylvestris* in mixed forest, Palacio de Valdellorma, Spain.

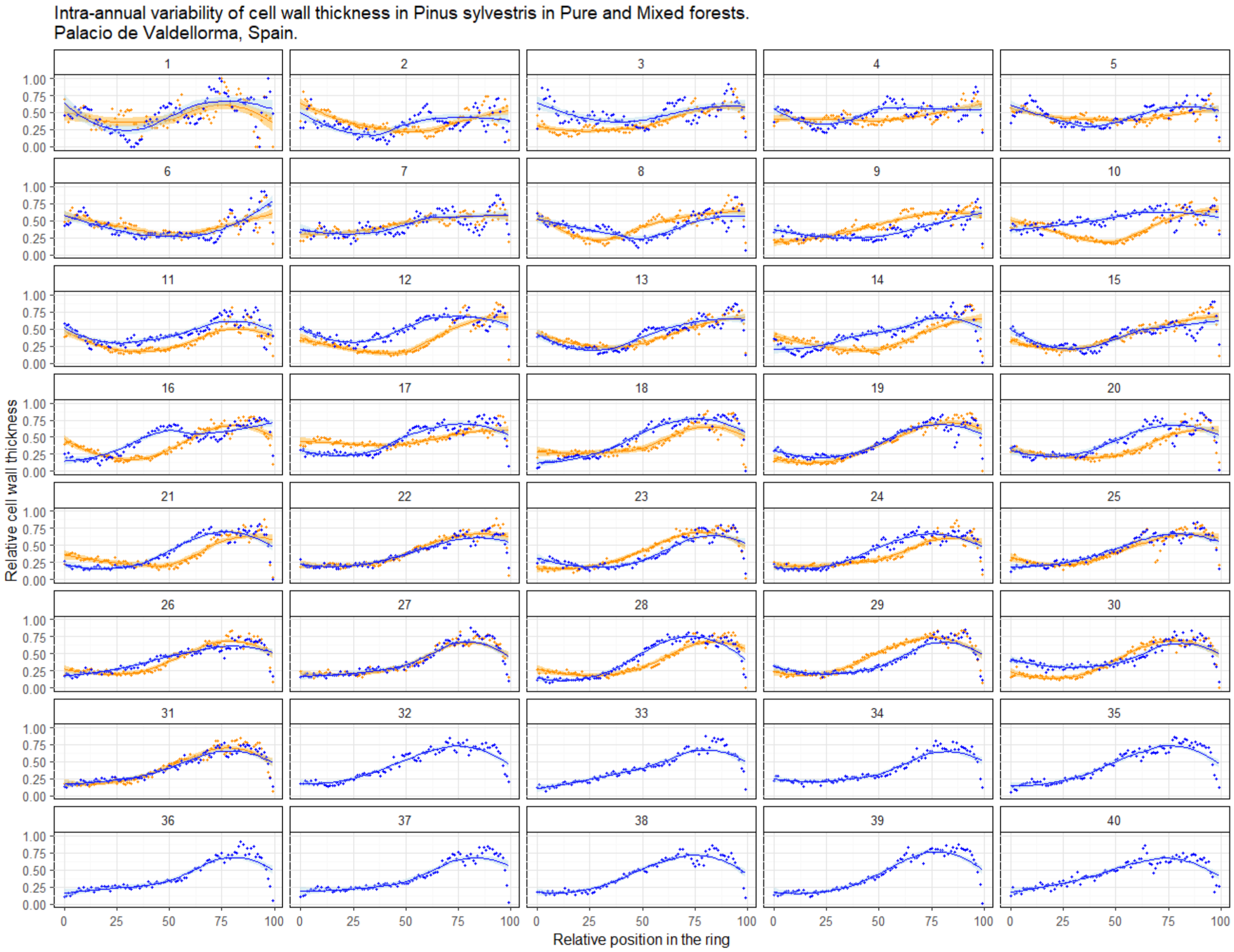

Supplementary Figure S3B Intra-annual profiles of cell wall thickness aggregated per forest type plotted along the growing season of each year following the ontogenetic development of trees. The intra-annual profiles are loess fit, the shaded area represents confidence interval set at 95%. Blue represents *P. sylvestris* in pure forest and orange *P. sylvestris* in mixed forest, Palacio de Valdellorma, Spain.

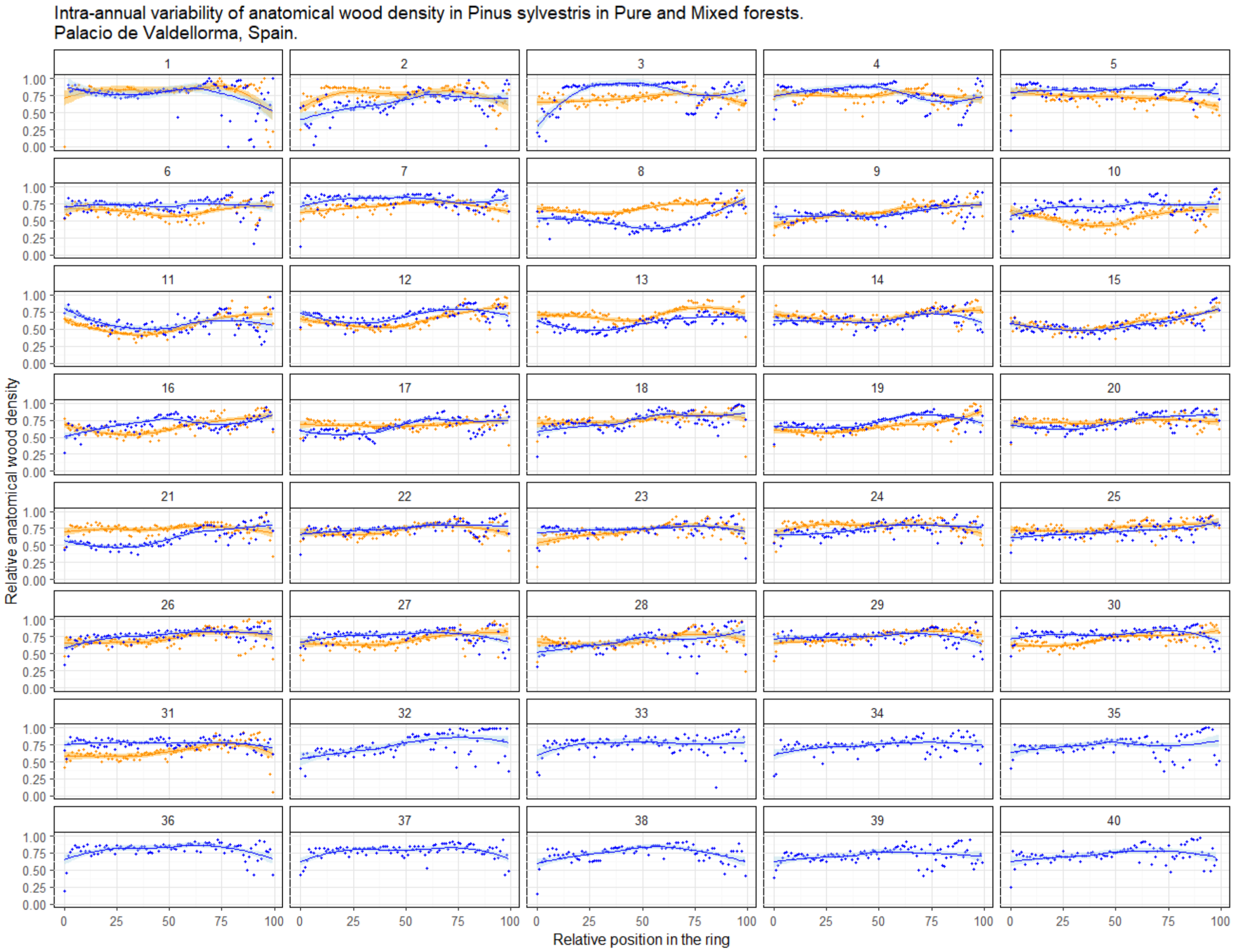

Supplementary Figure S3C Intra-annual profiles of anatomical wood density aggregated per forest type plotted along the growing season of each year following the ontogenetic development of trees. The intra-annual profiles are loess fit, the shaded area represents confidence interval set at 95%. Blue represents *P. sylvestris* in pure forest and orange *P. sylvestris* in mixed forest, Palacio de Valdellorma, Spain.

1.2 Supplementary Tables

Supplementary Table S1 Correlation function results between Monthly mean temperature (Mean T), monthly precipitation (P) and Standardised Precipitation Evapotranspiration Index calculated at six-month resolution (SPEI 6) and the following wood anatomy traits: Ring Width Index, (RWI) mean hydraulic diameter (DH), cell wall thickness (CWT), anatomical wood density (AD) of *P. sylvestris* in the pure and mixed forest in Poland (Rogow), timespan 1958 – 2018 and Spain (Palacio de Valdellorma), timespan 1990 – 2018. In this table, only significant correlations are reported.

SITE	Forest Type	Climatic variable	Month	coef	significant	Lower CI	Upper CI	EW-LW	Wood anatomical trait
POLAND	Mixed	Pre	Mar	-0.33622	TRUE	-0.54537	-0.09748	EW	DH
POLAND	Mixed	Pre	Mar	0.278456	TRUE	0.06754	0.474183	EW	AD
POLAND	Mixed	Pre	Mar	0.251405	TRUE	0.00695	0.472849	LW	DH
POLAND	Mixed	Pre	Apr	-0.31075	TRUE	-0.4851	-0.10839	LW	CWTALL
POLAND	Mixed	Pre	Jul	0.385823	TRUE	0.140889	0.589874	LW	RWI
POLAND	Mixed	Pre	Dec	-0.22168	TRUE	-0.41613	-0.02048	EW	AD
POLAND	Mixed	Pre	Dec	0.209087	TRUE	0.028576	0.375475	LW	CWTALL
POLAND	Mixed	Pre	Dec	0.320461	TRUE	0.111164	0.509295	LW	AD
POLAND	Mixed	Pre	FEB	0.276104	TRUE	0.009028	0.515054	LW	RWI
POLAND	Mixed	Pre	JUL	0.359545	TRUE	0.126592	0.570033	LW	RWI
POLAND	Mixed	SPEI_6	Apr	-0.23508	TRUE	-0.4142	-0.03993	EW	RWI
POLAND	Mixed	SPEI_6	May	-0.32422	TRUE	-0.50607	-0.10549	EW	RWI
POLAND	Mixed	SPEI_6	May	0.325062	TRUE	0.133396	0.508152	LW	AD
POLAND	Mixed	SPEI_6	Jun	0.283694	TRUE	0.067995	0.479981	LW	AD
POLAND	Mixed	SPEI_6	Jul	0.276476	TRUE	0.027475	0.473721	LW	RWI
POLAND	Mixed	SPEI_6	Aug	0.321675	TRUE	0.084989	0.50843	LW	RWI
POLAND	Mixed	SPEI_6	Sep	0.344678	TRUE	0.079066	0.558704	LW	RWI
POLAND	Mixed	SPEI_6	Oct	0.297613	TRUE	0.058418	0.513073	LW	RWI
POLAND	Mixed	SPEI_6	Nov	0.254495	TRUE	0.022584	0.464116	EW	RWI
POLAND	Mixed	SPEI_6	Nov	0.329582	TRUE	0.095116	0.519844	LW	RWI
POLAND	Mixed	SPEI_6	Dec	0.242261	TRUE	0.000199	0.451762	LW	RWI
POLAND	Mixed	SPEI_6	APR	-0.32178	TRUE	-0.52546	-0.10609	LW	RWI
POLAND	Mixed	SPEI_6	MAY	-0.31805	TRUE	-0.54576	-0.08006	LW	RWI
POLAND	Mixed	SPEI_6	JUN	-0.28023	TRUE	-0.49815	-0.02191	LW	RWI
POLAND	Mixed	SPEI_6	OCT	0.281569	TRUE	0.054282	0.485625	EW	CWTALL
POLAND	Mixed	Tmean	Apr	-0.28657	TRUE	-0.48955	-0.05436	LW	RWI
POLAND	Mixed	Tmean	Jun	-0.19776	TRUE	-0.40232	-0.00977	LW	AD
POLAND	Mixed	Tmean	Jul	0.277105	TRUE	0.042679	0.487619	LW	CWTALL
POLAND	Mixed	Tmean	Aug	0.330262	TRUE	0.129641	0.5075	EW	CWTALL
POLAND	Mixed	Tmean	Oct	-0.30158	TRUE	-0.56192	-0.03228	EW	RWI
POLAND	Mixed	Tmean	Nov	0.249836	TRUE	0.016738	0.457418	LW	DH
POLAND	Mixed	Tmean	Nov	-0.22415	TRUE	-0.43494	-0.00315	LW	AD
POLAND	Mixed	Tmean	Dec	-0.25973	TRUE	-0.4971	-0.01736	EW	CWTALL

POLAND	Mixed	Tmean	JAN	-0.26489	TRUE	-0.41767	-0.06711	LW	CWTALL
POLAND	Mixed	Tmean	FEB	0.253041	TRUE	0.01821	0.451451	LW	RWI
POLAND	Mixed	Tmean	MAR	0.339585	TRUE	0.126799	0.542814	LW	RWI
POLAND	Mixed	Tmean	MAY	-0.3169	TRUE	-0.52184	-0.04905	EW	RWI
POLAND	Mixed	Tmean	JUN	0.264881	TRUE	0.054821	0.466554	EW	CWTALL
POLAND	Mixed	Tmean	JUL	-0.31107	TRUE	-0.56589	-0.03301	LW	RWI
POLAND	Mixed	Tmean	SEP	0.295363	TRUE	0.035699	0.533906	EW	RWI
POLAND	Mixed	Tmean	OCT	-0.25538	TRUE	-0.42446	-0.07774	EW	RWI
POLAND	Pure	Pre	Mar	-0.29497	TRUE	-0.49819	-0.09544	EW	RWI
POLAND	Pure	Pre	Jul	-0.3148	TRUE	-0.46479	-0.12239	LW	CWTALL
POLAND	Pure	Pre	Aug	0.218158	TRUE	0.003984	0.362018	LW	CWTALL
POLAND	Pure	Pre	Oct	-0.51094	TRUE	-0.72177	-0.09024	LW	CWTALL
POLAND	Pure	Pre	FEB	0.291398	TRUE	0.010827	0.52981	LW	RWI
POLAND	Pure	Pre	MAY	-0.27199	TRUE	-0.48345	-0.03848	EW	RWI
POLAND	Pure	Pre	JUN	-0.25645	TRUE	-0.43711	-0.03804	LW	DH
POLAND	Pure	Pre	JUL	0.398325	TRUE	0.201661	0.581249	LW	RWI
POLAND	Pure	Pre	OCT	0.465641	TRUE	0.239103	0.648218	EW	CWTALL
POLAND	Pure	Pre	OCT	-0.2749	TRUE	-0.47768	-0.0196	EW	DH
POLAND	Pure	Pre	OCT	0.258197	TRUE	0.019906	0.461903	EW	AD
POLAND	Pure	Pre	OCT	0.33432	TRUE	0.138828	0.499595	EW	RWI
POLAND	Pure	Pre	OCT	0.249114	TRUE	0.000213	0.481518	LW	AD
POLAND	Pure	Pre	SEP	0.437889	TRUE	0.084342	0.738586	EW	AD
POLAND	Pure	SPEI_6	Mar	-0.2328	TRUE	-0.40901	-0.00422	EW	AD
POLAND	Pure	SPEI_6	May	-0.32246	TRUE	-0.52618	-0.11975	EW	RWI
POLAND	Pure	SPEI_6	Jun	-0.3462	TRUE	-0.54254	-0.1412	EW	RWI
POLAND	Pure	SPEI_6	Sep	0.311011	TRUE	0.044103	0.55701	LW	RWI
POLAND	Pure	SPEI_6	Nov	0.313936	TRUE	0.035818	0.515555	EW	AD
POLAND	Pure	SPEI_6	Nov	-0.28114	TRUE	-0.46977	-0.00713	LW	CWTALL
POLAND	Pure	SPEI_6	Dec	-0.24676	TRUE	-0.43783	-0.00711	LW	CWTALL
POLAND	Pure	SPEI_6	FEB	-0.3933	TRUE	-0.66424	-0.00186	EW	DH
POLAND	Pure	SPEI_6	FEB	0.397803	TRUE	0.003185	0.701481	EW	AD
POLAND	Pure	SPEI_6	FEB	0.298575	TRUE	0.073522	0.472315	LW	DH
POLAND	Pure	SPEI_6	APR	0.246593	TRUE	0.0179	0.431491	LW	CWTALL
POLAND	Pure	SPEI_6	APR	-0.30693	TRUE	-0.48931	-0.09523	LW	RWI
POLAND	Pure	SPEI_6	MAY	-0.30097	TRUE	-0.5843	-0.00181	EW	CWTALL
POLAND	Pure	SPEI_6	MAY	-0.38161	TRUE	-0.60652	-0.05968	EW	AD
POLAND	Pure	SPEI_6	MAY	-0.42795	TRUE	-0.60409	-0.21168	LW	RWI
POLAND	Pure	SPEI_6	JUN	-0.30385	TRUE	-0.55054	-0.01785	EW	AD
POLAND	Pure	SPEI_6	JUN	-0.44993	TRUE	-0.62862	-0.24874	LW	RWI
POLAND	Pure	SPEI_6	AUG	0.245291	TRUE	0.025096	0.441083	LW	CWTALL
POLAND	Pure	Tmean	Apr	0.324482	TRUE	0.116644	0.473664	EW	DH
POLAND	Pure	Tmean	Apr	-0.25242	TRUE	-0.43675	-0.065	LW	RWI

POLAND	Pure	Tmean	Jul	-0.2276	TRUE	-0.42923	-0.01305	EW	CWTALL
POLAND	Pure	Tmean	Jul	-0.26515	TRUE	-0.4649	-0.0418	EW	RWI
POLAND	Pure	Tmean	Aug	-0.28579	TRUE	-0.48906	-0.06175	EW	RWI
POLAND	Pure	Tmean	Sep	-0.26732	TRUE	-0.44717	-0.06834	EW	CWTALL
POLAND	Pure	Tmean	Sep	-0.23784	TRUE	-0.45778	-0.03953	EW	AD
POLAND	Pure	Tmean	Sep	-0.22382	TRUE	-0.40101	-0.03647	EW	RWI
POLAND	Pure	Tmean	Oct	-0.41462	TRUE	-0.60968	-0.15908	EW	RWI
POLAND	Pure	Tmean	Dec	0.275264	TRUE	0.087347	0.455905	EW	RWI
POLAND	Pure	Tmean	Dec	-0.22457	TRUE	-0.40135	-0.01503	LW	CWTALL
POLAND	Pure	Tmean	JAN	0.405952	TRUE	0.179122	0.57043	EW	RWI
POLAND	Pure	Tmean	JAN	0.311647	TRUE	0.050471	0.517786	LW	DH
POLAND	Pure	Tmean	MAR	0.315202	TRUE	0.113379	0.533179	EW	RWI
POLAND	Pure	Tmean	MAR	0.346461	TRUE	0.141482	0.536237	LW	RWI
POLAND	Pure	Tmean	APR	-0.26868	TRUE	-0.48939	-0.06844	EW	RWI
POLAND	Pure	Tmean	MAY	-0.52638	TRUE	-0.67515	-0.3104	EW	RWI
POLAND	Pure	Tmean	JUN	-0.25291	TRUE	-0.49466	-0.01008	EW	RWI
POLAND	Pure	Tmean	JUL	-0.28266	TRUE	-0.48995	-0.05135	LW	RWI
POLAND	Pure	Tmean	OCT	-0.31024	TRUE	-0.48975	-0.11032	EW	RWI
SPAIN	Mixed	Pre	Apr	-0.43927	TRUE	-0.70785	-0.06352	EW	RWI
SPAIN	Mixed	Pre	Jun	-0.39946	TRUE	-0.76989	-0.02857	EW	CWTALL
SPAIN	Mixed	Pre	Jun	0.370758	TRUE	0.038206	0.630032	EW	RWI
SPAIN	Mixed	Pre	Jun	-0.4733	TRUE	-0.72325	-0.04086	LW	AD
SPAIN	Mixed	Pre	Jul	-0.39868	TRUE	-0.63877	-0.06487	LW	DH
SPAIN	Mixed	SPEI_6	Jun	-0.43572	TRUE	-0.71078	-0.11307	LW	AD
SPAIN	Mixed	SPEI_6	Jul	-0.50846	TRUE	-0.74722	-0.20982	LW	AD
SPAIN	Mixed	SPEI_6	Aug	-0.43275	TRUE	-0.70622	-5.5E-05	LW	AD
SPAIN	Mixed	SPEI_6	Sep	-0.3613	TRUE	-0.61813	-0.03642	LW	RWI
SPAIN	Mixed	SPEI_6	FEB	0.317619	TRUE	0.02209	0.555428	EW	DH
SPAIN	Mixed	SPEI_6	MAY	0.360465	TRUE	0.018999	0.626416	LW	AD
SPAIN	Mixed	SPEI_6	JUN	0.289824	TRUE	0.030539	0.513533	LW	AD
SPAIN	Mixed	SPEI_6	AUG	0.343764	TRUE	0.017769	0.599315	EW	RWI
SPAIN	Mixed	SPEI_6	SEP	0.350527	TRUE	0.021883	0.646189	EW	RWI
SPAIN	Mixed	SPEI_6	OCT	0.340492	TRUE	0.010144	0.637444	EW	RWI
SPAIN	Mixed	Tmean	Mar	0.479152	TRUE	0.225664	0.653568	LW	AD
SPAIN	Mixed	Tmean	Apr	-0.33241	TRUE	-0.65622	-0.01119	EW	AD
SPAIN	Mixed	Tmean	Jul	-0.4292	TRUE	-0.64592	-0.17002	LW	AD
SPAIN	Mixed	Tmean	Sep	0.37911	TRUE	0.036921	0.611724	EW	AD
SPAIN	Mixed	Tmean	Oct	0.391372	TRUE	0.006182	0.650595	LW	AD
SPAIN	Mixed	Tmean	Dec	-0.31875	TRUE	-0.54478	-0.01652	LW	CWTALL
SPAIN	Mixed	Tmean	JAN	0.334981	TRUE	0.034211	0.558586	LW	CWTALL
SPAIN	Mixed	Tmean	APR	0.393125	TRUE	0.059548	0.603602	LW	DH
SPAIN	Mixed	Tmean	APR	0.410581	TRUE	0.102055	0.633103	LW	RWI
SPAIN	Mixed	Tmean	JUL	-0.27789	TRUE	-0.47516	-0.0062	EW	CWTALL

SPAIN	Mixed	Tmean	AUG	0.396336	TRUE	0.076027	0.70036	EW	AD
SPAIN	Mixed	Tmean	SEP	0.286194	TRUE	0.017655	0.55463	LW	RWI
SPAIN	Mixed	Tmean	OCT	-0.31671	TRUE	-0.59409	-0.00068	EW	RWI
SPAIN	Pure	Pre	May	-0.38722	TRUE	-0.65209	-0.09871	LW	AD
SPAIN	Pure	Pre	Jun	0.427765	TRUE	0.01622	0.725251	LW	RWI
SPAIN	Pure	Pre	Oct	-0.37864	TRUE	-0.64514	-0.03768	LW	RWI
SPAIN	Pure	SPEI_6	Mar	0.328298	TRUE	0.018366	0.57941	LW	AD
SPAIN	Pure	SPEI_6	Apr	0.394839	TRUE	0.066294	0.68673	LW	AD
SPAIN	Pure	SPEI_6	May	0.399776	TRUE	0.036391	0.642475	EW	AD
SPAIN	Pure	SPEI_6	Aug	0.319544	TRUE	0.00619	0.587148	EW	CWTALL
SPAIN	Pure	SPEI_6	Aug	-0.34644	TRUE	-0.61872	-0.0074	LW	CWTALL
SPAIN	Pure	SPEI_6	Sep	-0.35943	TRUE	-0.66438	-0.04483	LW	AD
SPAIN	Pure	SPEI_6	MAY	-0.34681	TRUE	-0.53571	-0.06392	EW	AD
SPAIN	Pure	SPEI_6	JUN	-0.36976	TRUE	-0.64626	-0.0216	EW	AD
SPAIN	Pure	SPEI_6	SEP	0.409232	TRUE	0.073388	0.691783	EW	RWI
SPAIN	Pure	SPEI_6	SEP	0.312087	TRUE	0.016712	0.568549	LW	RWI
SPAIN	Pure	Tmean	Mar	0.396357	TRUE	0.076812	0.633475	LW	DH
SPAIN	Pure	Tmean	May	-0.36097	TRUE	-0.59886	-0.06053	EW	AD
SPAIN	Pure	Tmean	Jun	-0.41667	TRUE	-0.63718	-0.09634	EW	RWI
SPAIN	Pure	Tmean	Jun	0.445423	TRUE	0.112324	0.677073	LW	AD
SPAIN	Pure	Tmean	Jul	0.350337	TRUE	0.049573	0.625798	EW	CWTALL
SPAIN	Pure	Tmean	Nov	0.568663	TRUE	0.265583	0.811538	LW	DH
SPAIN	Pure	Tmean	Dec	0.395752	TRUE	0.092483	0.62298	LW	DH
SPAIN	Pure	Tmean	JAN	-0.39193	TRUE	-0.63192	-0.07308	EW	CWTALL
SPAIN	Pure	Tmean	JAN	-0.37464	TRUE	-0.62647	-0.06564	EW	AD
SPAIN	Pure	Tmean	FEB	-0.35777	TRUE	-0.63472	-0.02797	LW	AD
SPAIN	Pure	Tmean	JUN	-0.46969	TRUE	-0.70376	-0.05354	EW	CWTALL
SPAIN	Pure	Tmean	JUN	-0.32928	TRUE	-0.54128	-0.05404	EW	RWI
SPAIN	Pure	Tmean	JUN	-0.43617	TRUE	-0.66318	-0.12832	LW	DH
SPAIN	Pure	Tmean	JUN	-0.46398	TRUE	-0.70201	-0.18136	LW	RWI
SPAIN	Pure	Tmean	AUG	0.276879	TRUE	0.033886	0.586362	LW	DH
SPAIN	Pure	Tmean	OCT	-0.58097	TRUE	-0.78908	-0.24995	EW	DH
SPAIN	Pure	Tmean	OCT	0.378533	TRUE	0.059457	0.613623	EW	AD

Supplementary Table S2 Kolmogorov-Smirnov test results between xylem traits profiles before, during and after drought events in *P. sylvestris* in mixed (Top) and pure forest (Bottom), in the site in Poland (left panel) and in the site in Spain (right panel). DH = mean hydraulic diameter, CWT = cell wall thickness, AD = anatomical wood density. Significant correlations are highlighted in bold.

**Site**	**Rogów**				**Spain**			
**Forest type / Wood anatomical traits**	**MIXED**							
**DH**	Drought	BEFORE	DURING	AFTER	**Drought**	**BEFORE**	**DURING**	**AFTER**
	BEFORE	-	-	-	**BEFORE**	-	**0.001**	-
	DURING	-	-	-	**DURING**	**0.001**	-	**0.01**
	AFTER	-	-	-	**AFTER**	-	**0.01**	-
**CWTALL**	Drought	BEFORE	DURING	AFTER	**Drought**	**BEFORE**	**DURING**	**AFTER**
	BEFORE	-	-	-	**BEFORE**	**-**	**0.001**	**-**
	DURING	-	-	-	**DURING**	**-**	**-**	**0.01**
	AFTER	-	-	-	**AFTER**	**-**	**0.01**	**-**
**AD**	**Drought**	**BEFORE**	**DURING**	**AFTER**	**Drought**	**BEFORE**	**DURING**	**AFTER**
	**BEFORE**	**-**	**-**	**0.05**	**BEFORE**	**-**	**0.001**	**-**
	**DURING**	**-**	**-**	**-**	**DURING**	**0.001**	**-**	**0.001**
	**AFTER**	**0.05**	**-**	**-**	**AFTER**	**-**	**0.001**	**-**
**Site**	**Rogów**				**Spain**			
**Forest type / Wood anatomical traits**	**PURE**							
**DH**	**Drought**	**BEFORE**	**DURING**	**AFTER**	**Drought**	**BEFORE**	**DURING**	**AFTER**
	**BEFORE**	**-**	**0.01**	**-**	**BEFORE**	**-**	**0.001**	**0.001**
	**DURING**	**0.01**	**-**	**-**	**DURING**	**0.001**	**-**	**0.001**
	**AFTER**	**-**	**-**	**-**	**AFTER**	**0.001**	**0.001**	**-**
**CWTALL**	Drought	BEFORE	DURING	AFTER	**Drought**	**BEFORE**	**DURING**	**AFTER**
	BEFORE	-	-	-	**BEFORE**	-	**0.007**	**0.03**
	DURING	-	-	-	**DURING**	**0.007**	-	-
	AFTER	-	-	-	**AFTER**	**0.03**	-	-
**AD**	**Drought**	**BEFORE**	**DURING**	**AFTER**	**Drought**	**BEFORE**	**DURING**	**AFTER**
	**BEFORE**	**-**	**-**	**0.01**	**BEFORE**	**-**	**0.03**	**0.001**
	**DURING**		**-**	**0.006**	**DURING**	**0.03**	**-**	**0.001**
	**AFTER**	**0.01**	**0.006**	**-**	**AFTER**	**0.001**	**0.001**	**-**
